# SUMOylation of the Forkhead Transcription Factor FOXL2 Promotes Its Stabilization/Activation through Transient Recruitment to PML Bodies

**DOI:** 10.1371/journal.pone.0025463

**Published:** 2011-10-12

**Authors:** Adrien Georges, Bérénice A. Benayoun, Mara Marongiu, Aurélie Dipietromaria, David L'Hôte, Anne-Laure Todeschini, Jana Auer, Laura Crisponi, Reiner A. Veitia

**Affiliations:** 1 Programme de Pathologie Moléculaire et Cellulaire, Institut Jacques Monod, UMR 7592 CNRS-Université Paris Diderot, Paris, France; 2 Université Paris-Diderot/Paris VII, Paris, France; 3 Ecole Normale Supérieure de Paris, Paris, France; 4 Istituto di Ricerca Genetica e Biomedica, Consiglio Nazionale delle Ricerche, Cagliari, Italy; 5 Université Paris-Sud/Paris XI, Orsay, France; 6 Faculté de Médecine Cochin-Port-Royal, Université Paris Descartes/Paris V, Paris, France; University of California Merced, United States of America

## Abstract

**Background:**

FOXL2 is a transcription factor essential for ovarian development and maintenance. It is mutated in the genetic condition called Blepharophimosis Ptosis Epicantus inversus Syndrome (BPES) and in cases of isolated premature ovarian failure. We and others have previously shown that FOXL2 undergoes several post-translational modifications.

**Methods and Principal Findings:**

Here, using cells in culture, we show that interference with FOXL2 SUMOylation leads to a robust inhibition of its transactivation ability, which correlates with a decreased stability. Interestingly, FOXL2 SUMOylation promotes its transient recruitment to subnuclear structures that we demonstrate to be PML (Promyelocytic Leukemia) Nuclear Bodies. Since PML bodies are known to be sites where post-translational modifications of nuclear factors take place, we used tandem mass spectrometry to identify new post-translational modifications of FOXL2. Specifically, we detected four phosphorylated, one sulfated and three acetylated sites.

**Conclusions:**

By analogy with other transcription factors, we propose that PML Nuclear Bodies might transiently recruit FOXL2 to the vicinity of locally concentrated enzymes that could be involved in the post-translational maturation of FOXL2. FOXL2 acetylation, sulfation, phosphorylation as well as other modifications yet to be discovered might alter the transactivation capacity of FOXL2 and/or its stability, thus modulating its global intracellular activity.

## Introduction

FOXL2 is a conserved protein expressed in periocular, ovarian and pituitary cells [1] [2]. FOXL2 belongs to the family of Forkhead transcription factors and is mutated in the Blepharophimosis Ptosis Epicanthus inversus Syndrome (BPES) [3], a genetic disorder characterized by eyelid malformations often associated with premature ovarian failure [4]. Mutations of *FOXL2* in cases of isolated premature ovarian failure have also been reported [5] [6]. It has recently been shown that somatic ablation of *Foxl2* induces transdifferentiation of adult mice ovaries into testis-like structures [7], which is coherent with previous observations from studies in knock-out mice [8] [9]. These studies show that FOXL2 is required during ovarian development and in the adult to maintain the identity of the ovarian somatic cells. Very recently, a somatic mutation of *FOXL2* (p. C134W) was found in >95% of ovarian granulosa-cell tumors [10]. Several high-throughput studies have revealed that FOXL2 is involved in the regulation of various cellular processes, including sterol/steroid metabolism, cell stress response, apoptosis and reactive oxygen species metabolism [11] [12] [13] [14].

We and others have previously shown that FOXL2 undergoes various post-translational modifications (PTM), including serine phosphorylation and SUMOylation [12] [15] [16] [17]. Moreover, overexpression of the histone deacetylase SIRT1 leads to alterations in the transactivation capacity of FOXL2, suggesting its acetylation [12]. FOXL2 appears to have various and sometimes antagonistic roles towards processes such as granulosa cells proliferation, differentiation and apoptosis [18]. It has thus been proposed that the variety of combinations of PTM isoforms might help explain how FOXL2 can specifically regulate such different processes [18] [19]. The study of the mechanisms of FOXL2 post-translational maturation might thus be of crucial interest to better understand ovarian organogenesis, function and neoplasia.

Members of the Small Ubiquitin-related MOdifier (SUMO) family of ubiquitin-like proteins act as PTM moieties on target proteins [20] [21]. Four SUMO isoforms have been described in vertebrates (SUMO1-4), and share substantial sequence homology [20]. Similarly to ubiquitin, SUMO proteins undergo cleavage after a diglycine motif by SUMO-activating enzymes (E1). Activated SUMO is then covalently linked to UBC9 (E2), the only SUMO-conjugating enzyme described thus far, and subsequently attached to the ε-amino group of target lysine residues [20]. A third enzyme (E3 ligase), forming a complex with SUMO-conjugated UBC9 and a SUMOylatable substrate, enhances the efficiency and specificity of SUMO transfer. Conjugation to SUMO is reversible and deSUMOylation is catalyzed by SUMO-specific peptidases (SENPs) [22].

SUMOylation of transcription factors leads mostly to transcriptional repression by participating to the recruitment of histone deacetylases (HDAC) and subsequent heterochromatinization of target genes, as well as the formation and recruitment of transcriptional repressor complexes [23] [24] [25]. In a few cases, SUMOylation has been reported to increase the activity of transcription factors, such as the heat shock factors HSF1 and HSF2 [26] [27], NFAT1 [28], SOX9 [29], JunB [30] and RORα [31].

SUMOylation has been associated to Promyelocytic Leukemia (PML) Nuclear Bodies (PML NBs), which are stable nuclear domains enriched in PML and SP100 proteins [32] [33]. Van Damme and collaborators have referenced more than 170 proteins recruited to PML NBs, with functional implications in cell cycle, apoptosis, tumorigenesis, regulation of transcription, DNA damage response, etc [34]. However, the precise molecular function of PML NBs remains poorly understood [35] [36] [37] [38].

We have previously shown the existence of a FOXL2 SUMO1-conjugate [12]. Two recent reports have confirmed FOXL2 SUMOylation and suggested that lysines 25, 114 and 150 may be major conjugation sites [16] [17]. Moreover, FOXL2 was also found to directly interact with UBC9 and with the E3 SUMO-protein ligase PIAS1. FOXL2 is known to repress the expression of the steroidogenic acute response (*StAR*) gene [39] and its SUMOylation increases the extent of repression [16] [17].

SUMOylation alters the long-term fate of the target proteins even though the SUMO moiety can be rapidly deconjugated [23] [20]. Here, we set out to explore the biological significance of FOXL2 SUMOylation and found it to enhance its transcriptional activity via its transient recruitment to PML NBs. These subnuclear compartments could act as ‘modification factories’ for FOXL2, by bringing FOXL2 to the vicinity of locally concentrated modification enzymes. We have indeed identified eight new sites of post-translational modifications of FOXL2 by mass spectrometry, further revealing the complexity of FOXL2 protein maturation.

## Results and Discussion

### SUMOylation of FOXL2 modulates its transactivation ability

**Figure 1:**
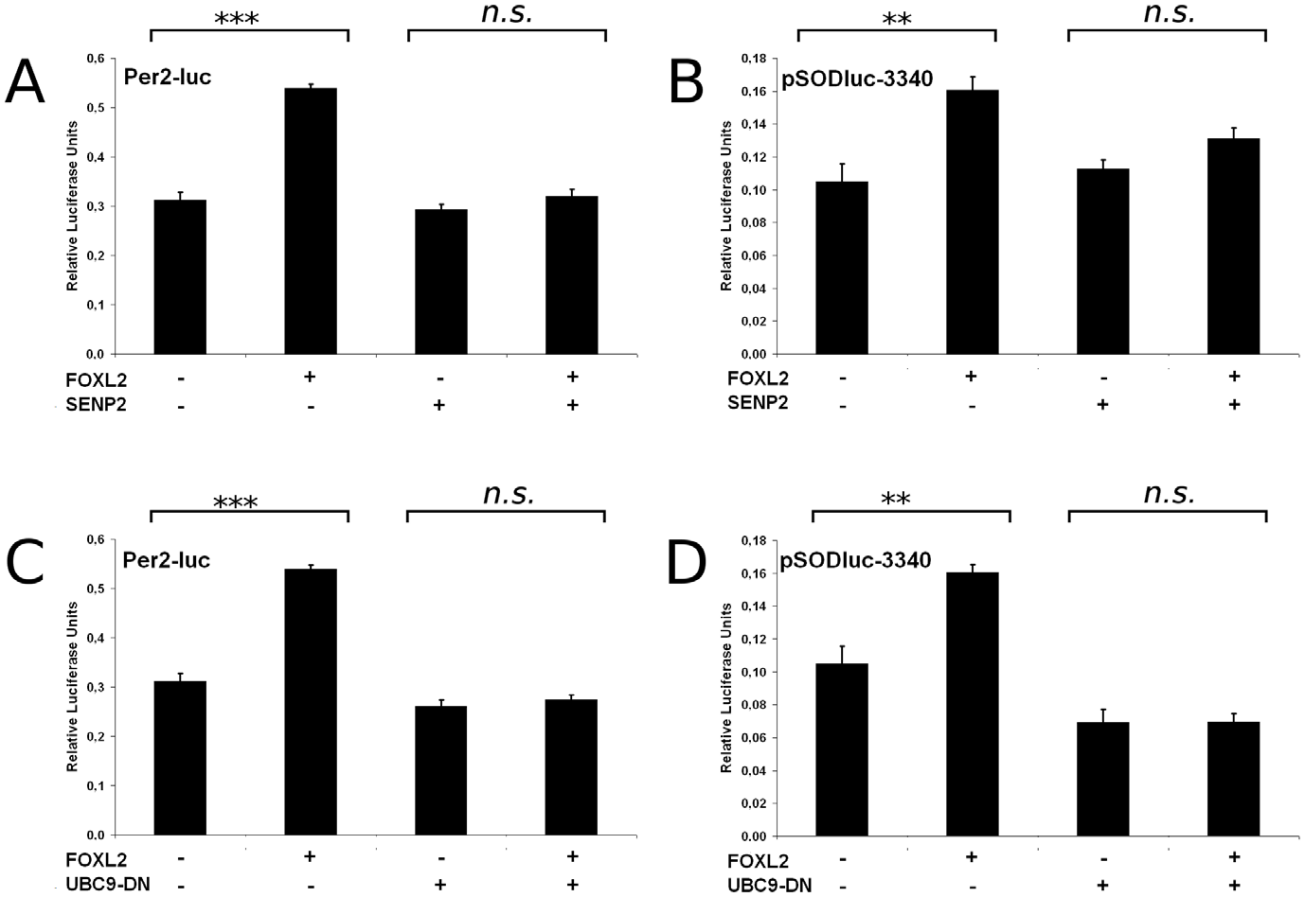
Interference with SUMOylation decreases FOXL2 transactivation. A and B) Luciferase assays in KGN cells transfected with Per2-luc and pSODluc-3340 (40 ng/well), respectively, with or without FOXL2 and SENP2 overexpression (each 20 ng/well). C and D) Luciferase assays in KGN cells transfected with Per2-luc and pSODluc-3340 (40 ng/well), respectively, with or without FOXL2 and UBC9-DN overexpression (each 20 ng/well). Each value is representative of six biological replicates. Error bars represent the standard error of the mean (SEM). Statistical significance in Student t-tests: n.s.: p>0.05, *: p<0.05, **: p<0.01, ***: p<0.001. All experiments were repeated at least three times with consistent results.

**Figure 2:**
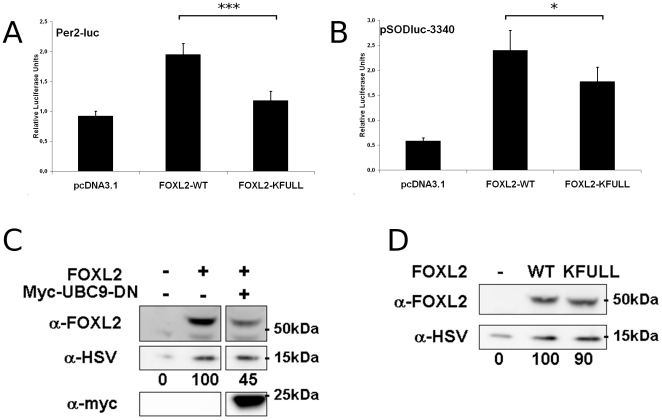
Enhanced stabilisation of FOXL2 partially explains the transcriptional effect of SUMOylation. A and B) Luciferase assays in KGN cells transfected with Per2-luc and pSODluc-3340 (40 ng/well), respectively, with or without FOXL2-WT or FOXL2-KFULL overexpression (20 ng/well). Each value is representative of six biological replicates. Error bars represent the SEM. Statistical significance in Student t-tests: n.s.: non-significant, *: p<0.05, **: p<0.01, ***: p<0.001 C) Western blot of whole cell extracts of KGN cells transfected with or without FOXL2-V5 and myc-UBC9-DN (each 1 ug/well in a 6 well plate), using pcDNA3.1 as a mock vector. Lic- (expressing a fusion protein His-Tag∶S-Tag∶HSV-Tag∶His-Tag) was used as an internal transfection control (500 ng/well). First panel: FOXL2. Second panel: Lic- (anti-HSV). Third panel: UBC9-DN (anti-myc). Below second panel is indicated the relative intensity of the FOXL2 band compared to the Lic- band. D) Western blot of whole cell extracts of KGN cells transfected for 24 h with or without FOXL2-WT-V5 or FOXL2-KFULL-V5 (2 µg/well in a 6-well plate). Lic- (500 ng) was used as a transfection control. First panel: FOXL2. Second panel: Lic- (anti-HSV). Below second panel is indicated the relative intensity of the FOXL2 band compared to the Lic- band. All experiments were repeated at least three times with consistent results.

We have previously shown that FOXL2-SUMO1 conjugates are endogenously present in granulosa and granulosa-like cells (AT29C and KGN cells) and pituitary-derived αT3 cells [12]. Here, in order to explore the functional role of FOXL2-SUMO1 conjugates and their products, we first measured the impact on FOXL2 activity of an interference with the global SUMOylation process in human granulosa-like KGN cells [40]. Specifically, we measured variations of the transactivation ability of transfected FOXL2 in response to either overexpression of the SUMO-deconjugating enzyme SENP2 or inhibition of SUMO-conjugation through the expression of a dominant-negative form of UBC9 (UBC9-DN; [41]). We assessed the activity of FOXL2 through its effect on the promoter of the genes *SOD2* and *Per2*. *SOD2* (or *MnSOD*) codes for an enzyme involved in the metabolism of reactive oxygen species (ROS), whereas *Per2* is a member of the Period gene family and is expressed in a circadian pattern. Both genes are directly regulated by FOXL2 in KGN cells [12] [42] and have previously been linked to tumor suppression [43] [44]. Interestingly, SENP2 and UBC9-DN overexpression both strongly decreased FOXL2 transactivation on the reporters pSODluc-3340 and Per2-luc (Fig. 1). These results suggest that SUMOylation of FOXL2 is required to ensure a normal transcriptional activity, but do not exclude the possibility of indirect effects (e.g. deSUMOylation of other transcription factors). A previous study showed that no FOXL2 SUMOylation was detectable in transfected COS-7 cells when lysine residues 25, 87, 114 and 150 of FOXL2 were mutated into arginines ([17], FOXL2-KFULL construct). We therefore compared the transactivation ability of this mutant to the one of wild-type FOXL2 (FOXL2-WT). Promoter activation by FOXL2-KFULL was indeed significantly lower than the activation elicited by FOXL2-WT, with a more pronounced effect on the *Per2* promoter (Fig. 2A–B). However, the transactivation ability of the KFULL mutant remained higher than what we observed when we overexpressed, for example, UBC9-DN. This might mean that SUMOylation increases FOXL2 activity through both direct and indirect ways, or that the KFULL mutant can still undergo some SUMOylation, though at a hardly detectable level.

Previous studies using the KFULL mutant showed that transrepression by FOXL2 on the *Steroidogenic Acute Response (StAR)* promoter was also enhanced by SUMOylation [16] [17]. To rationalize that both activation and repression are modulated by SUMOylation, we hypothesize that SUMOylation may increase the stability of FOXL2. To test this idea in our conditions, we overexpressed FOXL2 alone or in combination with UBC9-DN in KGN cells. Cells were cotransfected with a control vector driving the expression of a non-relevant HSV-tagged 15 kDa protein (referred to as “Lic-“) to check the efficiency of transfection (Fig. 2C). As expected, overexpression of UBC9-DN led to a 50% decrease of the FOXL2 protein level, which is to be correlated with its absence of transactivation ability in these conditions. However, a contribution of a modification of FOXL2 properties such as reduced DNA-binding or intrinsinc transactivation capacity cannot be excluded. Indeed, the stability of the KFULL mutant in KGN cells was only slightly lower than that of the WT (Fig. 2D), despite a marked difference in their transactivation abilities. This suggests that a modification of the intrinsic protein properties contributes to the loss of transactivation when SUMOylation is impaired.

### SUMOylation of FOXL2 induces its localization to subnuclear structures

**Figure 3:**
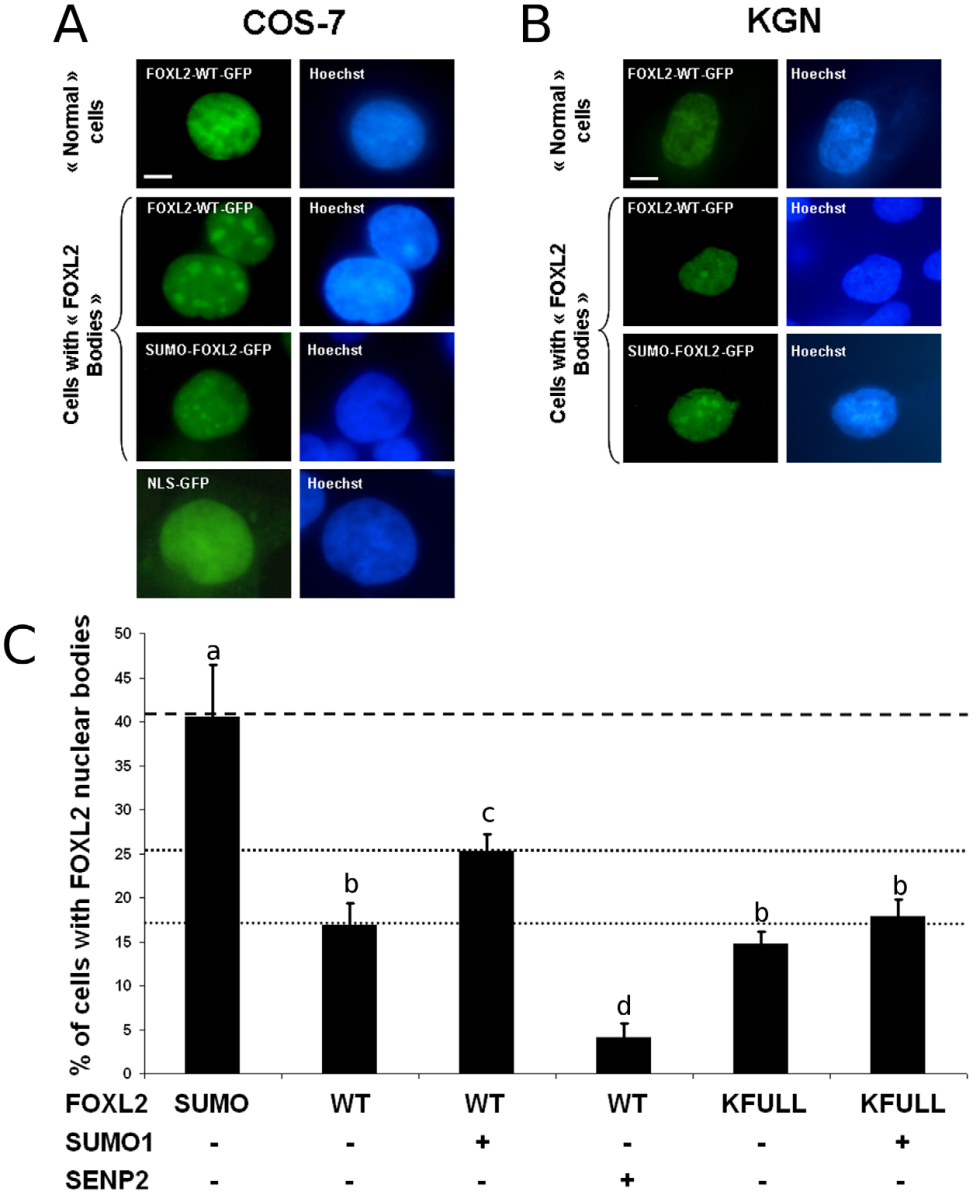
SUMOylation promotes FOXL2 recruitment to subnuclear structures. A and B) Presence of nuclear structures displaying high FOXL2-GFP concentration in a climate of high SUMOylation. Micrographs of COS-7 cells (A) and KGN cells (B) expressing FOXL2-GFP, the tripartite SUMO-FOXL2-GFP fusion or NLS-GFP, as indicated. Left column: GFP. Right column: Hoechst 33342 DNA staining. First line shows a representative “normal” cell, where FOXL2 displays its most common localisation, superimposable to DNA. Second and third lanes show representative cells with “FOXL2 nuclear bodies” where FOXL2 is enriched in subnuclear structures clearly distinct from chromatin structures. Fourth lane (only in A) shows a typical cell stained with nuclear localised GFP. Scale bar: 5 µm. Scale bar is valid for all micrographs. (C) Quantification of cells presenting subnuclear structures in COS-7 expressing SUMO-FOXL2-GFP, FOXL2-WT-GFP or FOXL2-KFULL-GFP with or without overexpression of SUMO1 or SENP2. At least 300 GFP-positive cells were scanned per condition (in groups of 50 to estimate the standard deviations). Error bar: SEM. Letters a, b, c, d refer to statistical categories in a Student's t-test. Conditions with different letters are statistically different with p<0.05.

To investigate whether SUMOylation had an impact on the subcellular distribution of FOXL2, we generated a construct driving the overexpression of a tripartite fusion protein involving, from N- to C-terminus, human SUMO1, FOXL2 and the GFP (hereafter called SUMO-FOXL2-GFP). Next, we transfected the FOXL2-GFP vector alone (control), FOXL2-GFP along with a SUMO1 expression vector, and our SUMO-FOXL2-GFP fusion vector into COS-7 cells, which have been extensively used to follow FOXL2 subcellular localization owing to their easy transfection [45] [46] [47]. Interestingly, transfection of the tripartite fusion construct led to high proportions of cells displaying subnuclear structures containing high local concentrations of GFP-fused proteins (Fig. 3A). These structures were also observed in some cells transfected with the FOXL2-GFP-expressing construct, but never in cells transfected with a NLS-GFP construct, where only GFP is expressed and localized to the nucleus. This shows that the presence of these structures does not result from a bias induced by the GFP tag (Fig. 3A). To verify that this localization of SUMOylated FOXL2 was not cell-line specific, we also overexpressed FOXL2-GFP or SUMO-FOXL2-GFP in KGN cells, in which we conducted the functional studies. As expected, FOXL2-GFP was mainly diffuse in the nucleus, whereas SUMO-FOXL2-GFP was enriched in subnuclear domains similar to those observed in COS-7 cells (Fig. 3B).

To further demonstrate that SUMOylation of FOXL2 promotes its recruitment to these structures, we expressed FOXL2-GFP alone or in combination with SUMO1 or SENP2, and determined the percentage of COS-7 cells displaying FOXL2 nuclear bodies (Fig. 3C). As expected, we observed an increase of the proportion of cells displaying nuclear bodies when SUMO1 was overexpressed from 15 to 25%, and a reduction when SENP2 is overexpressed, from 15 to less than 5%. More than 40% of cells transfected with the tripartite fusion construct displayed visible nuclear bodies. We also compared cells transfected with FOXL2-WT or FOXL2-KFULL mutant in fusion with the GFP, alone or with SUMO1. When FOXL2 (WT or KFULL) was expressed alone, about 15% of transfected cells displayed these bodies. However, when SUMO1 was co-overexpressed, the proportion of cells with visible nuclear bodies increased to 25% only for cells expressing FOXL2-WT, whereas there was no significant increase among KFULL expressing cells (Fig. 3C). This suggests that FOXL2 might be recruited to these structures independently of its SUMOylation (see discussion below) but also shows that FOXL2 SUMOylation enhances the frequency of appearance of these bodies.

It is worth mentioning that we failed to observe any obvious cytoplasmic localization of FOXL2-WT-GFP, FOXL2-KFULL-GFP or the single point mutants (K25R, K87R, K114R, K150R; data not shown). This suggests that the amount of cytoplasmic protein remains below our detection level, which also suggests that the lowered activity of the FOXL2-KFULL mutant noticed above is not due to a trivial mislocalization of FOXL2.

### FOXL2 is transiently recruited to PML Nuclear Bodies (PML NBs)

To explore whether or not these NBs contained diffusible or immobilized FOXL2 and to rule out any potential aggregation, we assessed FOXL2 mobility using Fluorescence Recovery After Photobleaching (FRAP) in COS-7 cells. Specifically, we transfected these cells with either FOXL2-GFP, FOXL2-GFP and SUMO1, or the vector expressing the tripartite fusion protein SUMO1-FOXL2-GFP. We then determined the time required to recover 50% of the maximum fluorescence (t1/2) after photobleaching, which is inversely proportional to protein mobility. For each cell, we bleached both a nucleoplasmic region and a nuclear structure enriched in FOXL2 (when relevant), to assess a potentially differential mobility (Fig. S1). We found that FOXL2-GFP alone displayed an average t1/2 of 9.3 s (±1.7 s), a value compatible with what we have previously reported [47]. This value was not significantly different from that of GFP-fused proteins in the nucleoplasmic portions of cells co-expressing FOXL2-GFP and SUMO1 or the tripartite fusion protein (respectively 10.3 s±4.3 s and 7.2 s±1.7 s). Recovery of fluorescence did not show statistically significant differences of protein mobility the nuclear structures of the co-transfected cells and of cells transfected with the tripartite fusion construct (respectively 6.8 s±1.4 s and 5.2 s±0.8 s).

**Figure 4:**
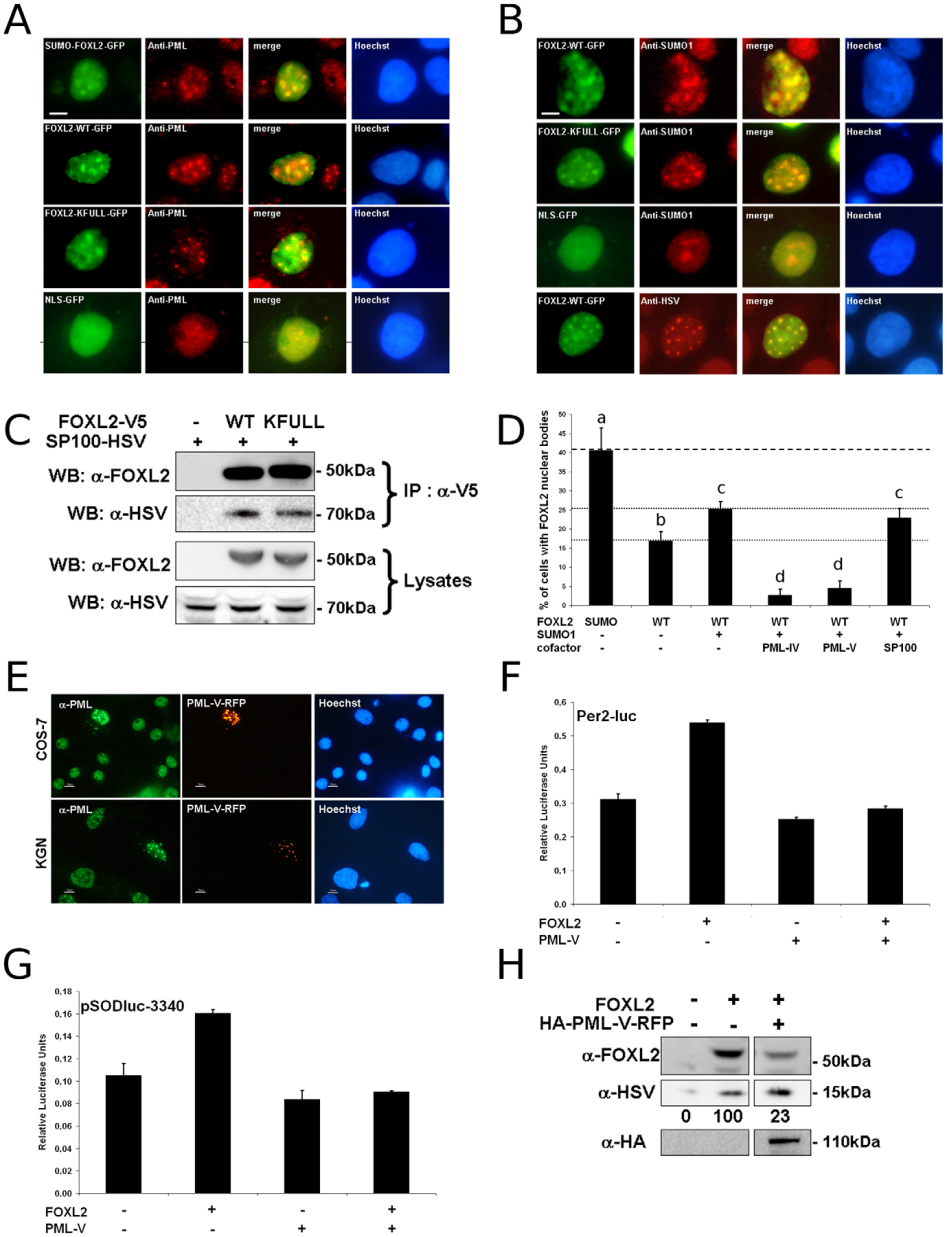
FOXL2 recruitment to PML Bodies enhances its transactivation ability. A and B) FOXL2 colocalization with PML, SUMO1 and SP100 in nuclear bodies. COS-7 cells overexpressing SUMO-FOXL2-GFP, FOXL2-WT-GFP, FOXL2-KFULL-GFP or NLS-GFP (as indicated, green color) were stained with an anti-PML antibody (panel A, red) or an anti-SUMO1 anti-body (panel B, first three lines, red) In the fourth line of panel B, COS-7 cells overexpressing FOXL2-WT-GFP and SP100A-HSV were stained with an anti-HSV antibody. Representative cells with FOXL2 enriched in nuclear bodies are shown. DNA was stained using Hoechst 33342 (in blue). Scale bar: 5 µm. Scale bar is valid for all micrographs. (C) Interaction between FOXL2 and SP100. SP100-HSV was expressed alone (lane 1) or co-expressed with V5-tagged FOXL2-WT (lane 2) or KFULL (lane 3). FOXL2 was precipitated using anti-V5-conjugated affinity resin for western blot analysis. Upper panel shows a 50 kDa band detected with anti-FOXL2 antibody. Second panel shows a 70 kDa band detected with an anti-HSV antibody. Third and fourth panels display a western blot analysis of the lysates, with anti-FOXL2 and anti-HSV antibodies, respectively. (D) Quantification of cells displaying subnuclear structures in COS-7 expressing SUMO-FOXL2-GFP or FOXL2-WT-GFP with or without overexpression of SUMO1, PML-IV-RFP, PML-V-RFP or SP100A-HSV. At least 300 GFP-positive cells were scanned per condition (in groups of 50 to estimate the standard deviations). Error bar: SEM. Letters a, b, c, d refer to statistical categories in a Student's t-test. Conditions with different letters are statistically different with p<0.05. (E) PML localisation in COS-7 or KGN cells overexpressing PML-V-RFP. Green: anti-PML directed against all isoforms of PML. Red: PML-V-RFP. Blue: DNA stained with Hoechst 33342. In non-transfected cells, PML is mostly nucleoplasmic with a few dots (1–5 in COS-7 cells, 5–20 in KGN cells) indicative of PML NBs. With PML-V-RFP expressed, PML NBs are much enlarged and the proportion of nucleoplasmic PML is decreased. Scale bar: 10 µm. Scale bar is valid for all micrographs. F and G) Luciferase assays in KGN cells transfected with Per2-luc and pSODluc-3340 (40 ng/well), respectively, with or without FOXL2 and PML-V-RFP overexpression (each 20 ng/well). Each value is representative of six biological replicates. Error bars represent SEM. Statistical significance in Student t-tests: n.s.: p>0.05, *: p<0.05, **: p<0.01, ***: p<0.001. H) Western blot of whole cell extracts of KGN cells transfected with or without FOXL2-V5 and PML-V-RFP (2 µg/well in a 6-well plate), using pcDNA3.1 as a mock vector. Lic- (expressing a fusion protein His-Tag∶S-Tag∶HSV-Tag∶His-Tag) was used as a transfection control (500 ng). First panel: FOXL2. Second panel: Lic- (anti-HSV). Third panel: PML-V (anti-HA). Below second panel is indicated the relative intensity of the FOXL2 band compared to the Lic- band. All experiments were repeated at least three times with consistent results.

To further characterize the subnuclear structures enriched in SUMOylated FOXL2, we performed immunofluorescence experiments on COS-7 cells co-transfected with FOXL2-GFP and SUMO1 expression vectors with antibodies recognizing well-characterized subnuclear structures. No colocalization was observed with components of nuclear speckles, transcription factories, gems or Cajal bodies (Fig. S2) (see refs. [48] [49] [50] for reviews on these structures). Interestingly, SUMO FOXL2 bodies were stained by an anti-PML antibody (Fig. 4A). These structures being PML NBs is coherent with the observation that they are also enriched in SUMO1 (Fig. 4B). Interestingly, we have performed a FOXL2 two-hybrid screen using a mouse ovary cDNA library which retrieved a potential interaction between FOXL2 and the E3 SUMO-protein ligase PIAS2 (2 prey clones), the SUMO-conjugating enzyme UBC9 (2 prey clones) and the nuclear autoantigen SP100, which is an organizer of PML-NBs (2 clones). These results suggest that PIAS2 is involved in FOXL2 SUMOylation and confirm previous findings on the interaction between FOXL2 and UBC9 [16]. At the same time the latter finding lends credence to our two-hybrid screening. Next, we decided to further validate the interaction between FOXL2 and SP100. We therefore coexpressed FOXL2-GFP with SUMO1 and an HSV-tagged version of SP100A (the most abundant form of SP100). Immunodetection of SP100 showed that it colocalized with FOXL2, further emphasizing that the latter is recruited to PML NBs (Fig. 4B). Furthermore, SP100 could be co-immunoprecipitated with FOXL2 (either WT or KFULL, Fig. 4C), suggesting that a direct interaction with SP100 may be a way to recruit FOXL2 to PML NBs. We tried to co-immunoprecipitate endogenous PML with FOXL2 but failed to find any direct interaction (not shown). A plausible explanation for this result is that only SUMOylated FOXL2 may interact with the SUMO-binding domain of PML. To further study the mode of recruitment of FOXL2 to PML NBs, we overexpressed SP100 or two isoforms of PML (IV and V) tagged with RFP along with FOXL2 and SUMO1 and counted the number of cells displaying NBs (Fig. 4D). Overexpression of SP100 had no marked effect on FOXL2 localization or PML NBs organization, except a marginal reduction of the proportion of cells with NBs. This probably means that endogenous SP100 is not a limiting factor for PML NBs formation and FOXL2 recruitment. Surprisingly, overexpression of either PML isoform almost fully abrogated localization of FOXL2 to PML NBs (i.e. 25% to 3–4% of cells). No colocalization of FOXL2 and these PML isoforms could be observed (not shown). Furthermore, PML NBs generally seemed disorganized, enlarged and the amount of soluble PML seemed lower than in non-transfected cells (Fig. 4E). This might be due to non-functional aggregation of PML proteins due to an excess of protein or to an imbalance between isoforms. Indeed, it has been shown that expression different PML isoforms in a PML null background led to very different PML NBs, suggesting that expression of specific isoforms may influence the formation and structure of PML NBs [51]. Based on this result, we decided to test the effect of the overexpression of PML-V on FOXL2 transactivation ability. We found that it completely abolished the activation by FOXL2 of either *SOD2* or *Per2* promoters (Fig. 4F–G). Furthermore, the stability of FOXL2 was severely impaired by PML-V overexpression (Fig. 4H). These results suggest that well-organized PML NBs are necessary for the stabilization/activation of FOXL2. These results taken together allow us to propose that the effect of FOXL2 SUMOylation is mainly to promote its recruitment to PML NBs.

### Mapping of the post-translational modifications of FOXL2 by mass spectrometry

**Figure 5:**
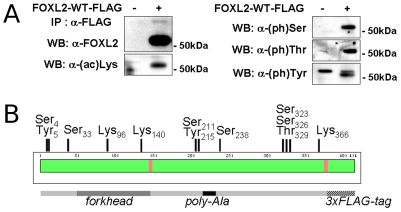
FOXL2 is acetylated and phosphorylated on multiple residues. A) Western blot following anti-FLAG immunoprecipitation of COS-7 lysates and SDS-PAGE. Cells were transfected with a mock vector (pcDNA3.1) or FOXL2-FLAG. Independent immunoblots were performed using anti-FOXL2 C-terminus antibody, mouse anti-acetyl-Lysine (Cell Signaling Technologies), mouse anti-phospho-Serine, anti-phospho-Threonine and anti-phospho-Tyrosine (Sigma-Aldrich), as indicated on the left of each image. B) Schematic representation of FOXL2 post-translational modifications identified by mass spectrometry. Immunoprecipitated FOXL2, in the form of a crude eluate or the isolated 51–52 kD band revealed by Coomasie staining, was digested using trypsin or chymotrypsin and analyzed using ESI-MS-MS with an Orbitrap analyser. Databases were searched with acetylation of lysines and phosphorylation of serines, threonines and tyrosines set as dynamic modification in addition of modification of cysteines by a carbamidomethyl or n-ethylmaleimide groups and oxidation of methionine that were due to sample preparation. FOXL2 sequence is represented by a green and red bars, the green color representing residues present in at least one high confidence peptide, whereas other residues are in red. The positions of the phosphorylated and acetylated residues in the FOXL2 sequence, based on the database search, are represented. Manual inspection revealed that the modification on serine 4 or tyrosine 5 is more likely a sulfation than a phosphorylation. Lower bar represents the position of the forkhead and poly-Alanine domains.

SUMOylation is a transient modification, affecting a small percentage of a particular protein at a time. We therefore hypothesized that SUMOylation, which mediates FOXL2 recruitment to PML NBs, might also mediate its further post-translational processing, which would explain the alteration of FOXL2 activity observed when SUMOylation is impaired. Here, we have mapped some of these PTMs by mass spectrometry, in order to test if non-modifiable mutants may recapitulate the inactivation of FOXL2 observed through inhibition of its SUMOylation. We performed an immunoprecipitation of FLAG-tagged FOXL2 transiently overexpressed in COS-7 cells, whose easy transfection allowed us to recover the required amount of protein. We first tested the presence of specific modifications of FOXL2 in our samples by Western blot using antibodies targeting phosphorylated serine, threonine and tyrosine residues and acetylated lysines (Fig. 5A). All antibodies recognized a 51–52 kDa band when FOXL2 was immunoprecipitated but not in the control, showing that FOXL2 undergoes these modifications. We then performed tandem mass spectrometry on FOXL2 peptides, using an Orbitrap analyzer. To maximize sequence coverage, we used both tryptic and chymotryptic treatments and we used both direct digestion of affinity purified FOXL2, which avoids the biases due to SDS-PAGE and in-gel digestion. At least two independent analyses were conducted for each digestion mode. Databases were searched using SEQUEST and Mascot engines, with phosphorylation and acetylation set as dynamic modifications. We obtained an excellent coverage (97%) of the FOXL2 sequence (Fig. 5B), with multiple matches for most peptides (Table S1). Many phosphorylated peptides were identified by both search engines in multiple spectra (Fig. 5B, Table 1 and Table 2). Serine 33 was independently found in 7 types of peptides with a +80 Da mass shift, with one peptide where it was the only phosphorylatable residue (Table 1, Table 2 and Fig. S3). Peaks typical of phosphorylation, corresponding to the loss of a H_3_PO_4_ group (neutral loss of 98 Da) from the precursor were observed in the MS/MS spectrum and confirmed the attribution. Serine 238 was found in several peptides that could be unambiguously attributed due to the neighboring polyalanine domain that has a characteristic fragmentation pattern (Table 1, Table 2 and Fig. S4). Peptides were found with and without a +79.966 mass shift characteristic of phosphorylation and serine 238 was the only phosphorylatable residue in these peptides. Some fragments with a 98 Da neutral loss were also visible. A third class of phosphorylated peptides included serine 203, 211 and tyrosine 215 (Table 1, Table 2 and Fig. S5). Peaks corresponding to the loss of one phosphate group from the precursor could be observed but had a very low intensity, whereas peaks still bearing the phosphate group were present. Serine 203 could be ruled out as the phosphorylated residue because y-ion series clearly allowed us to locate the phosphorylation near the C-terminus of the peptide. However serine 211 and tyrosine 215 could not be discriminated. The last class of phosphorylated peptides included serine residues 323, 326 and 339 as well as threonine 329 and 338 (Table 1, Table 2 and Fig. S6). Non phosphorylated fragments were visible in the MS/MS spectra, but the neutral loss was only partial and peaks bearing the 80 Da modification were present. The b-series of ions clearly allowed the localization of the phosphorylation site near the N-terminus of the peptide. However, the peptide (SPASPATAA) containing serines 323 and 326 and threonine 329 was poorly fragmented, and the exact position of the phosphorylation could not be assigned.

**Table 1:** Peptides detected as modified by a phosphorylation, sulfation or acetylation using Mascot.

Peptide sequence	Modification	Modified residue	digestion	matches	highest Score	charge
APETGRTVKEPEGPPPSPGKGGGGGGGTAPEKPDPAQKPPY	**Phosphorylation**	**Serine 33**	Chymotrypsin	3	**39**	+5
APETGRTVKEPEGPPPSPGKGGGGGGGTAPEKPDPAQKPPYSY	**Phosphorylation**	**Serine 33**	Chymotrypsin	16	**43**	+5
LAPETGRTVKEPEGPPPSPGKGGGGGGGTAPEKPDPAQKPPYSY	**Phosphorylation**	**Serine 33**	Chymotrypsin	4	**39**	+4
EPEGPPPSPGK	**Phosphorylation**	**Serine 33**	Trypsin	20	**50**	+2
TVKEPEGPPPSPGK	**Phosphorylation**	**Serine 33**	Trypsin	13	**65**	+2
LNNSWPLPQPPSPMPY	**Phosphorylation**	**Serine 211/Tyrosine 215**	Chymotrypsin	5	**27**	+2
NNSWPLPQPPSPMPY	**Phosphorylation**	**Serine 211/Tyrosine 215**	Chymotrypsin	7	**31**	+2
AScQMAAAAAAAAAAAAAAGPGSPGAAAVVKGL	**Phosphorylation**	**Serine 238**	Chymotrypsin	6	**50**	+3
HAAAAPPPAPPHHGAAAPPPGQLSPASPATAAPPAPAPTSAPGLQF	**Phosphorylation**	**Serine 323/Serine 326/Threonine 329**	Chymotrypsin	7	**48**	+3
MMASYPEPEDAAGAL	**Sulfation**	**Serine 4/Tyrosine 5**	Chymotrypsin	9	**37**	+2
MMASYPEPEDAAGALL	**Sulfation**	**Serine 4/Tyrosine 5**	Chymotrypsin	7	**35**	+2
MMASYPEPEDAAGALLAPETGR	**Sulfation**	**Serine 4/Tyrosine 5**	Trypsin	10	**61**	+3
KGWQNSIR	**Acetylation**	**Lysine 96**	Trypsin	2	**65**	+2

Each line represents a group of matches reaching our quality cut-off corresponding to the same peptide sequence with the indicated modification. Mascot matches were selected to have a peptide score >20. Detail of all peptide matches (modified and unmodified) obtained by both search engines is available as supplementary information (Table S1).

**Table 2:** Peptides detected as modified by a phosphorylation, sulfation or acetylation using SEQUEST.

Peptide sequence	Modification	Modified residue	digestion	matches	highest Xcorr	charge
APETGRTVKEPEGPPPSPGKGGGGGGGTAPEKPDPAQKPPY	**Phosphorylation**	**Serine 33**	Chymotrypsin	1	**4,53**	+5
APETGRTVKEPEGPPPSPGKGGGGGGGTAPEKPDPAQKPPYSY	**Phosphorylation**	**Serine 33**	Chymotrypsin	2	**4,74**	+5
APETGRTVKEPEGPPPSPGKGGGGGGGTAPEKPDPAQKPPYSYVAL	**Phosphorylation**	**Serine 33**	Chymotrypsin	3	**5,12**	+4
LAPETGRTVKEPEGPPPSPGKGGGGGGGTAPEKPDPAQKPPYSY	**Phosphorylation**	**Serine 33**	Chymotrypsin	2	**5,04**	+4
LAPETGRTVKEPEGPPPSPGKGGGGGGGTAPEKPDPAQKPPYSYVAL	**Phosphorylation**	**Serine 33**	Chymotrypsin	2	**5,06**	+4
EPEGPPPSPGK	**Phosphorylation**	**Serine 33**	Trypsin	33	**2,66**	+2
TVKEPEGPPPSPGK	**Phosphorylation**	**Serine 33**	Trypsin	24	**4,14**	+2
LNNSWPLPQPPSPMPY	**Phosphorylation**	**Serine 211/Tyrosine 215**	Chymotrypsin	27	**3,94**	+2
NNSWPLPQPPSPMPY	**Phosphorylation**	**Serine 211/Tyrosine 215**	Chymotrypsin	16	**3,41**	+2
AScQMAAAAAAAAAAAAAAGPGSPGAAAVVKGL	**Phosphorylation**	**Serine 238**	Chymotrypsin	5	**6,21**	+3
AAAAAAAAAAAAAAGPGSPGAAAVVKGL	**Phosphorylation**	**Serine 238**	Chymotrypsin	19	**7,47**	+3
AAAAAAAAAAAAAAGPGSPGAAAVVKGLAGPAASY	**Phosphorylation**	**Serine 238**	Chymotrypsin	6	**6,67**	+3
HAAAAPPPAPPHHGAAAPPPGQLSPASPATAAPPAPAPTSAPGLQF	**Phosphorylation**	**Serine 323/Serine 326/Threonine 329**	Chymotrypsin	5	**4,45**	+3
SPASPATAAPPAPAPTSAPGLQF	**Phosphorylation**	**Serine 323/Serine 326/Threonine 329**	Chymotrypsin	5	**3,38**	+2
MMASYPEPEDAAGAL	**Sulfation**	**Serine 4/Tyrosine 5**	Chymotrypsin	8	**2,47**	+2
MMASYPEPEDAAGALL	**Sulfation**	**Serine 4/Tyrosine 5**	Chymotrypsin	1	**2,19**	+2
MMASYPEPEDAAGALLAPETGR	**Sulfation**	**Serine 4/Tyrosine 5**	Trypsin	12	**4,74**	+3
ASYPEPEDAAGAL	**Sulfation**	**Serine 4/Tyrosine 5**	Chymotrypsin	17	**3,38**	+2
ASyPEPEDAAGALL	**Sulfation**	**Serine 4/Tyrosine 5**	Chymotrypsin	8	**2,61**	+2
KGWQNSIR	**Acetylation**	**Lysine 96**	Trypsin	4	**2,2**	+2
TLDPACEDMFEKGNY	**Acetylation**	**Lysine 140**	Chymotrypsin	1	**2,63**	+2
DHDSKTGALHSRLDQGQF	**Acetylation**	**Lysine 366**	Chymotrypsin	1	**3,17**	+2

Each line represents a group of matches reaching our quality cut-off corresponding to the same peptide sequence with the indicated modification. SEQUEST matches were selected to have a XCorr>2 when peptide charge was +2, >2.5 with peptide charge +3, >3 with peptide charge +4, and >4 with peptide charge >+5. Detail of all peptide matches (modified and unmodified) obtained by both search engines is available as supplementary information (Table S1).

Another apparent phosphorylation site was identified at the N-terminus of the protein, either at serine 4 or tyrosine 5 (Table 1, Table 2 and Fig. S7). However, no neutral loss corresponding to phosphorylation could be observed in the MS/MS spectra. Moreover, in the group of spectra attributed to the peptides having this modification, the precursor molecular weight had an average difference of −5.5 ppm with respect to the predicted mass, whereas the average difference for all the peptides was 0.1 ppm, with a standard deviation of only 1.2 ppm. This was specific to the presence of the modification, as peptide matches attributed to a peptide including serine 4 and tyrosine 5 and not having a +80 Da modification had a measured mass corresponding to the predicted mass. Two chymotryptic peptides (ASYPEPEDAAGAL and ASYPEPEDAAGALL) were identified with and without the modification, allowing direct calculation of its mass based on the measured precursor masses. The masses retrieved were 79.956+/−0.003 Da and 79.957+/−0.001 Da respectively. The only simple modification fitting this pattern is sulfation (or sulfonation), which has mainly been described for tyrosines, but also detected on serines and threonines [52] [53]. This modification usually produces a neutral loss of 80 Da in the fragmentation spectra that unfortunately could not be observed in this case. However, the b-ion series allowed us to map the 80 Da modification to the SYPEPE part of the fragment. This subfragment does not fragment correctly in the presence of the modification. Moreover, doubly-charged b-ions seemed favored when the modification was present, compared to singly-charged b-ions, suggesting that intramolecular hydrogen bonds involving the sulfate group may retain positive charges and stabilize the N-terminal fragment.

Acetylated peptides were less abundantly identified than phosphorylated peptides, but we found several potentially modified peptides (Table 1 and Table 2). Indeed, comparison of fragmentation spectra with spectra of the corresponding peptides without acetylation (identified with high reliability) confirmed the existence of a +42.010 Da moiety on the relevant peptides. The first peptide involved lysine 96 (Fig. S8). It was only found in one of the four conditions, but 2 spectra were attributed with good scores in each of the replicates. Other acetylated peptides were detected by only one match and involve lysine 140 (Fig. S9) and 366 (Fig. S10), respectively.

**Figure 6:**
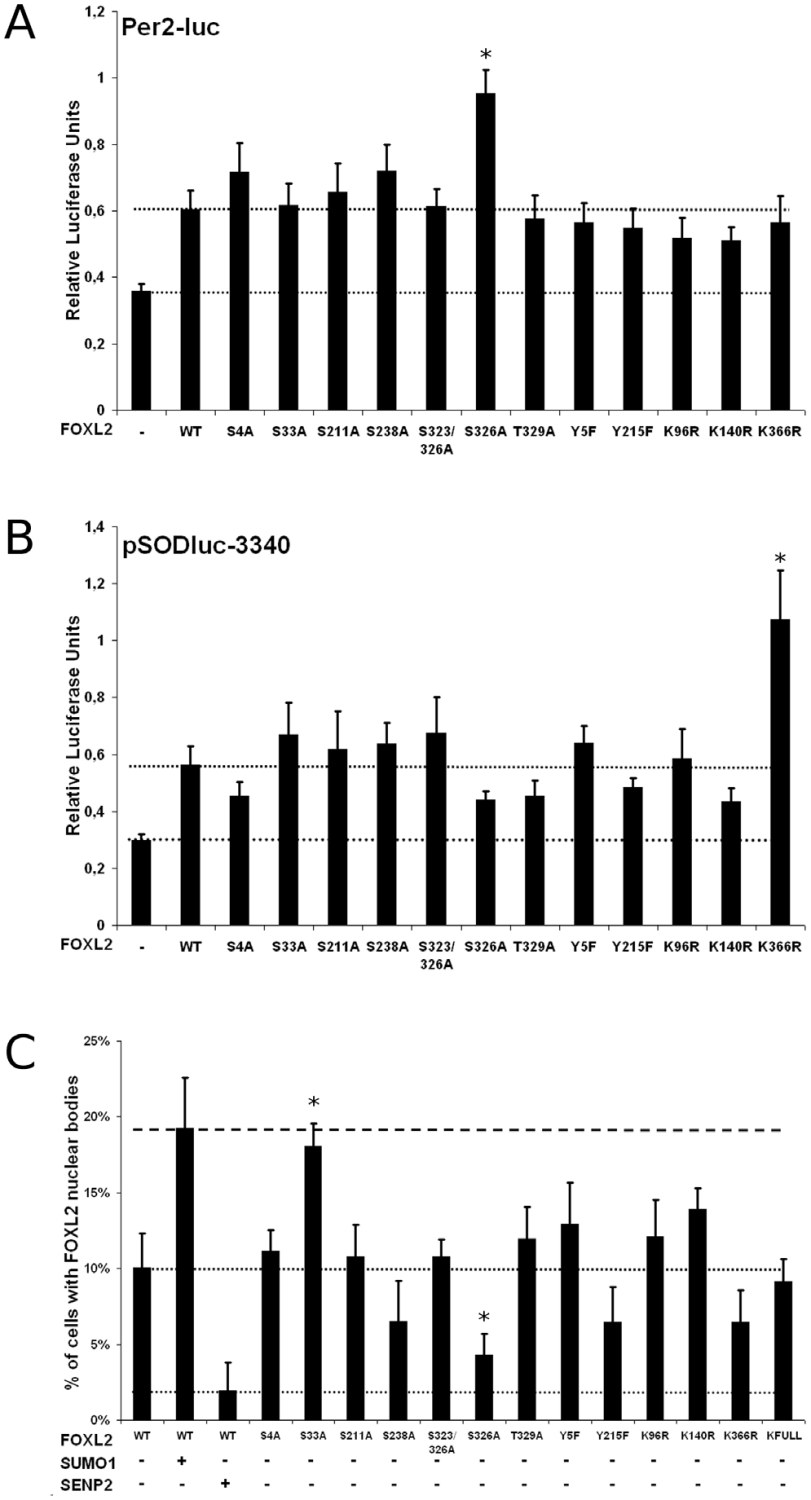
Transactivation ability and localisation rate to PML NBs of non-modifiable mutants. A and B) Luciferase assays in KGN cells transfected with Per2-luc and pSODluc-3340 (40 ng/well), respectively, with or without overexpression of wild-type FOXL2 or various FOXL2 alleles (20 ng/well), each one corresponding to a substitution of a potentially modified residue by an amino-acid simulating the non-modified residue. Each value is representative of six biological replicates. Error bars represent the SEM. Statistical significance in Student t-tests, compared to FOXL2-WT: *: p<0.05. Hyperactivities of FOXL2-S326A on Per2-luc and FOXL2-K366R on pSODluc-3340 compared to FOXL2-WT were seen only in one experiment out of four. C) Quantification of cells presenting subnuclear structures in COS-7 expressing wild-type FOXL2-GFP with or without overexpression of SUMO1 or SENP2, or the same FOXL2-GFP alleles as above. At least 200 GFP-positive cells were scanned per condition (in groups of at least 50 cells to estimate the standard deviations). Error bar: SEM. Statistical significance in Student t-tests, compared to FOXL2-WT: *: p<0.05.

We next generated variants of FOXL2 bearing a mutation at each one of the modified or potentially modified residues, in fusion with the GFP. Mutations were designed to represent the characteristics of the non-modified version of FOXL2, and thus a (potentially) modified serine/threonine residue was replaced by an alanine, a tyrosine by a phenylalanine and a lysine by an arginine. We generated isolated mutants for each residue except for serine 323 which was mutated along with serine 326, predicted as a stronger phosphorylation site. We tested the transactivation ability of such mutants on the *Per2* and *SOD2* promoters and analyzed their subcellular localization in COS-7. Surprisingly, on the *Per2* promoter, all mutants were as active as FOXL2-WT (Fig. 6A). A mild hyper-activation of FOXL2-S326A was detected in some experiments but was highly variable. On the *SOD2* promoter, some mutants tended to be slightly less active than FOXL2-WT, but this difference was not statistically significant (pmin = 0.2, for S238A) (Fig. 6B). Again, a mild hyper-activation of FOXL2-K366R was detected in some experiments but was variable from one to another.

Coherently with these results, the subcellular localization of the mutants was very similar to the localization of FOXL2-WT. No cytoplasmic localization or aggregation could be observed in any of the mutants. In addition, all mutants were able to localize to at NBs (colocalizing with PML), similar to the ones observed with the WT protein. Interestingly, the frequency of appearance of the nuclear bodies was, however, altered for some mutants (Fig. 6C). Indeed, FOXL2-S33A recruitment to NBs was as high as FOXL2-WT recruitment to PML NBs when SUMO1 was overexpressed (20% compared to 10% without SUMO1 overexpression). Conversely, FOXL2-S326A was significantly less present in NBs than FOXL2-WT expressed alone and not significantly different from FOXL2-WT along with SENP2 overexpression. Other mutants tended to be less present in PML NBs (S238A, Y215F, K366R), but without statistical support. These results suggest that phosphorylation at serines 33 and 326 may play a role in FOXL2 recruitment to or transient retention at PML NBs. As mutation of these residues does not have a significant effect on FOXL2 transactivation ability, it seems that multiple and combinatorial post-translational modifications are necessary to explain the long term effects of SUMOylation. More extensive studies will be necessary to decipher the precise role of each modification and the functional interactions that may occur among multiple modifications. The mechanism by which SUMOylation stimulates FOXL2 transactivation remains largely unknown but our work shows that a moderate stabilization and localization to the PML NBs are likely to be part of this mechanism.

### Conclusions

Here, we find that SUMOylation targets FOXL2 to PML NBs. Interference with the SUMOylation machinery or PML NB formation leads to a decrease of FOXL2 activity on two target promoters potentially related to the role of FOXL2 in ROS metabolism and tumor suppression. SUMO1-fused FOXL2 is highly present in PML NBs and interference with SUMOylation decreases the proportion of cells where FOXL2 colocalizes with PML NBs. Interestingly, a direct interaction with the PML NB scaffold protein SP100 may contribute to SUMO-independent FOXL2 recruitment to these subnuclear structures.

PML NBs are enriched in a number of enzymes involved in the PTM of proteins, including kinases, HATs, HDACs, SUMO- and ubiquitin-ligases and methyl-transferases (Table 3). Thus, post-translational maturation of FOXL2 would be a logical consequence of its SUMOylation. Thus, we tried to map as much post-translational modifications of FOXL2 as possible. We detected two phosphorylation sites on serines 33 and 238, one either on serine 211 or tyrosine 215, and at least one in the cluster composed of serines 323, 326 and threonine 329. Interestingly, the GPS 2.1 tool predicts the six serine/threonine sites to be potential targets of the CMGC group of kinases [54] [55], which includes cyclin-dependent kinases, mitogen-activated protein kinases, dual specificity tyrosine-phosphorylation-regulated kinases and glycogen-synthase kinases. Phosphorylation at serine-proline (SP) sites could thus be a major aspect of FOXL2 post-translational modification. Interestingly, LATS1, which is the only kinase known to interact with FOXL2 thus far, also interacts with CDK1 to arrest early mitosis [56]. Phosphorylation of FOXL2 might then be cell-cycle dependent and might in turn affect the modulation of cell cycle by FOXL2. Phosphorylation at tyrosine 215 would also be very interesting if confirmed. Indeed, the naturally-occuring mutant pTyr215Cys is one of the very few disease-causing substitutions in *FOXL2* sequence located outside the forkhead domain [57]. Further studies beyond the scope of this work will be necessary to better understand the role of these phosphorylations.

**Table 3:** Components of the post-translational machinery present in PML NBs.

Type of modification	Protein name	Uniprot accession number	effect
**Acetylation**	**CBP**	*Q92793*	**+**
	**p300**	*Q09472*	**+**
	**TIP60**	*Q92993*	**+**
	**HDAC1**	*Q13547*	**−**
	**HDAC2**	*Q92769*	**−**
	**HDAC3**	*O15379*	**−**
	**HDAC7**	*Q8WUI4*	**−**
	**SIRT1**	*Q96EB6*	**−**
**Methylation**	**PRMT1**	*Q99873*	**+**
	**EZH2**	*Q15910*	**+**
**Phosphorylation**	**CK1**	*P48729*	**+**
	**CK2**	*P68400*	**+**
	**MNK2b**	*Q9HBH9*	**+**
	**p38**	*Q16539*	**+**
	**IKKe**	*Q14164*	**+**
	**ATR**	*Q13535*	**+**
	**ATM**	*Q13315*	**+**
	**mTOR**	*P42345*	**+**
	**STK6**	*O14965*	**+**
	**CHK2**	*O96017*	**+**
	**HIPK1**	*Q86Z02*	**+**
	**HIPK2**	*Q9H2X6*	**+**
	**HIPK3**	*Q9H422*	**+**
	**ZIPK**	*O43293*	**+**
**SUMOylation**	**SUMO-1**	*P63165*	
	**SUMO-2**	*P61956*	
	**SUMO-3**	*P55854*	
	**UBA2**	*Q9UBT2*	**+**
	**UBC9**	*P63279*	**+**
	**PIASy**	*Q8N2W9*	**+**
	**RANBP2**	*P49792*	**+**
	**MMS21**	*Q96MF7*	**+**
	**SENP1**	*Q9P0U3*	**−**
	**SENP2**	*Q9HC62*	**−**
	**SENP5**	*Q96HI0*	**−**
**Ubiquitination**	**TOPORS**	*Q9NS56*	**+**
	**BRCA1**	*P38398*	**+**
	**RNF6**	*Q9Y252*	**+**
	**RBCK1**	*Q9BYM8*	**+**
	**MDM2**	*Q00987*	**+**
	**E6AP**	*Q05086*	**+**
	**FBX3/SCF**	*Q9UK99*	**+**
	**CULLIN1**	*Q13616*	**+**
	**SKP1**	*P63208*	**+**
	**PLZF**	*Q05516*	**+**
	**UBP7**	*Q93009*	**−**

Enzymes involved in post-translational modifications of proteins and described as associated with PML Nuclear Bodies, according to Van Damme *et al.* [34]. These proteins represent about 30% of all proteins associated with PML NBs, highlighting a potential integrative role of PML NBs in post-translational maturation of nuclear proteins. Proteins followed by a + sign are mainly involved in the transfer of post-translational moieties to protein substrates (e.g. kinases), whereas those with a − sign are associated to protein demodification (e.g. phosphatases). Uniprot accession IDs are in italic.

The precision of the Orbitrap analyser also allowed us to detect the potential sulfation of either serine 4 or tyrosine 5. The functional meaning of sulfation on FOXL2 remains to be studied. Indeed, tyrosine sulfation usually affects extracellular and secreted proteins [58]. However, it has been reported as an extremely abundant modification, which may suggest the existence of substrates in various subcellular compartments. Serine/threonine O-sulfation of a nuclear receptor has previously been described [59], but very few studies have focused on this modification thus far. Finally, we gathered evidence for acetylation on three lysine residues, although the depth of detection was rather poor. Interestingly, lysine 96 belongs to a consensus (K/RxKK) found in many p300 targets [60] and we have previously shown that p300 overexpression stimulates FOXL2 transactivation [12]. However, all these PTMs, at least studied separately, had very little effect on FOXL2 transactivation ability. Thus,the functional effects of their combinations deserve further attention.

PML NBs could play the role of catalysts for FOXL2 maturation by recruiting it to the vicinity of modifying enzymes. However, little is known about the process of FOXL2 SUMOylation itself. The presence of a phosphorylation site after lysine 25, evoking a phospho-dependent SUMOylation motif, may suggest a role for FOXL2 phosphorylation in its SUMOylation. Further studies extending our results on the PTMs of FOXL2 and their potential modulation of its interaction with protein partners will be of great interest.

Until now, SUMOylation of transcription factors has been mostly associated with transcription repression, mainly through recruitment of general transrepressor complexes that possess SUMO interacting motifs [24]. However, a few exceptions have been found where SUMO-conjugation is crucial to enhance the transactivation ability of several transcription factors. Based on our findings concerning FOXL2, we propose that SUMOylation may be instrumental for maturation of transcription factors through recruitment to ‘modification factories’, including PML NBs.

## Materials and Methods

### Ethics statement

Given that this study deals only with established cell lines approval by an ethics committee was not required.

### Mammalian cell expression and reporter vectors

The luciferase reporters pSODluc-3340 and Per2-luc (a generous gift from Pr. Gad Asher) are pGL3-basic vectors, and have been described previously [61] [62]. Plasmid DNA for SENP2 expression vector was a kind gift from Pr. Yanping Zhang [63]. The UBC9-DN mammalian expression vectors were a gift from Pr. Yin-Yuan Mo [41]. The FOXL2-GFP expression vector was described previously, and KFULL-GFP was created using the same protocol [47]. PML-IV-RFP and PML-V-RFP were kind gifts from Dr Annie Sittler and Pr Richard Day, respectively [64]. FOXL2-FLAG was created by replacing the GFP coding region by a 3XFLAG cassette using the XbaI and Kpn2I sites present in the previous construct. The FOXL2-V5 and KFULL-V5 constructs were created using the pcDNA3.1/V5-His TOPO TA Expression Kit (Invitrogen) according to manufacturer's instructions. The FOXL2-GFP N-terminal SUMO1 fusion (SUMO1-FOXL2-GFP) was obtained by junction PCR as described previously [47], using pFOXL2-GFP and a PCR-amplified human SUMO1 coding region, and subsequent cloning in frame with the GFP ORF in pCDNA3.1/CT-GFP (Invitrogen). SP100A was amplified from pooled cDNAs from cultured cells (HeLa, Jurkat, K562, KGN and COV434) and inserted in frame with an HSV tag into pTriex4 Ek/LIC vector (Novagen), according to the manufacturer's instructions. Lic- (expressing a fusion protein His-Tag∶S-Tag∶HSV-Tag∶His-Tag) was created by inserting a short DNA sequence (10 bp) in frame with the tags of pTriex-4 Ek/LIC vector.

### Yeast two-hybrid screening

The yeast two-hybrid screen using the FOXL2 bait was carried out by Dualsystems Biotech AG, Zurich, Switzerland. The bait construct for yeast two-hybrid screening was made by subcloning a full length FOXL2 cDNA into the vector pLexA-DIR (Dualsystems Biotech AG, Zurich, Switzerland). We used a mutated version of FOXL2 (H104N) that is hypomorphic and does not lead to aggregation, in order to avoid the toxicity displayed by the wild-type protein in yeast. The bait construct was transformed into the strain NMY32 (MATa his3D200 trp1-901 leu2-3,112 (lexAop)8-ADE2 LYS2::(lexAop)4-HIS3 URA3::(lexAop)8-lacZ GAL4) using standard procedures. Correct expression of the bait was verified by western blotting of cell extracts using a mouse monoclonal antibody directed against the LexA domain (Dualsystems Biotech, Switzerland). The absence of self-activation was verified by co transformation of the bait together with a control prey and selection on minimal medium lacking the amino acids tryptophan, leucine and histidine (selective medium). For the yeast two-hybrid screen, the bait was cotransformed together with a mouse ovary cDNA library (8.106 independent clones) into NMY32. Positive transformants were tested for beta-galactosidase activity using a PXG b-galactosidase assay (Dualsystems Biotech) and when positive were considered to be true interatactants. Library plasmids were isolated from positive clones. The identity of positive interactors was determined by sequencing.

### Cell culture and transient transfections

Granulosa-like KGN cells were used to perform functional analysis as they are close to ovarian granulosa cells where FOXL2 is normally expressed. They were grown in DMEM-F12 medium, supplemented with 10% FBS and 1% penicillin/streptomycin (Invitrogen-Gibco). COS-7 cells were used in experiments where high level of transfection were required (to score protein localization or in immunoprecipitation assays). They were grown in DMEM medium supplemented with 10% FBS and 1% penicillin/streptomycin. Cells were seeded 16 h prior to transfection at a density of 4×10^4^ cells.cm^−2^ (KGN cells) or 2.5×10^4^ cells.cm^−2^ (COS-7 cells), and transfected using the calcium-phosphate method (COS-7) or Lipofectamine 2000 reagent (KGN) (Invitrogen) [65].

### Luciferase assays

Luciferase assays were performed in KGN cells as previously described [47]. Cells seeded at 50% confluence in 96-well plates were transfected with 40 ng/well reporter, 5 ng/well transfection control and 20 ng/well of each plasmid using Lipofectamine 2000 reagent (Invitrogen) (1 uL/well), then lysed 24 h after transfection and analyzed with the Dual-Glo Luciferase Assay System (Promega) according to manufacturer's instruction. Relative luciferase units values given for all experiments are the means of values obtained from 6 biologically independent replicates, and represent the ratio of firefly luciferase activity over Renilla luciferase activity in the samples. Statistical significance was estimated by a Student t-test. Errors bars represent the Standard Deviation.

### Immunoprecipitation and Antibodies

COS-7 cells were lysed 48 h after transfection in Lysis Buffer (50 mM Tris, 150 mM NaCl, 1 mM EDTA, 1% Triton X-100, pH 7.6) supplemented with protease inhibitors (PMSF 0.1 mM and Complete mini EDTA-free cocktail, Roche), phosphatase inhibitors (PhosSTOP, Roche), deacetylase inhibitors (TSA 1 µM and nicotinamide 5 mM) and N-ethylmaleimide 12,5 mM. Clarified lysates were immunoprecipitated using anti-FLAG M2 magnetic beads (Sigma) according to manufacturer's instructions. Precipitated proteins were eluted in 50 mM Tris 150 mM NaCl pH 7.6 and 150 ng/uL 3XFLAG peptide (Sigma). Eluates were then separated by SDS-PAGE using NuPage Bis-Tris 4–12% Gels and MOPS-SDS Running Buffer (Invitrogen) and proteins were electrotransfered onto PVDF membranes (Hybond-P, GE Healthcare). The C-terminal peptide anti-FoxL2 polyclonal rabbit antibody has been described previously [1]. Monoclonal anti-acetylated lysine antibody was purchased from Cell Signaling technologies and used at a 1/1000 dilution. For tandem mass spectrometry, eluted proteins were separated by SDS-PAGE and stained with coomassie. The 50 kDa major band and eluate were both digested with trypsin or chymotrypsin (Sigma) according to the manufacturer's instruction.

### Tandem mass spectrometry

Digested peptides were separated on a nanoLC column using a 70 min gradient (for in gel digestion) and a 90 min gradient (for eluate digestion) and analysed in a LTQ Orbitrap Velos spectrometer (Thermo Fisher Scientific) with CID fragmentation. MS/MS spectra were analyzed with Mascot and Sequest, using a human database based on the Uniprot Knowledge Base, with a precursor match preference of 10 ppm and a fragment match tolerance of 0.8 Da. Modifications initially included in the research were phosphorylation of serines/threonines/tyrosines (+79.966 Da), acetylation of lysines (+42.010 Da), oxidation of methionines (+15.994 Da) and modification of cysteines with N-ethylmaleimide (+125.048 Da) or Carboxyamidomethyl (+57.021 Da). Peptides identified with FDR<0.01 by both engines were selected. Peptides identified only by one of the engines (including lysine 140 acetylation) were manually inspected and validated.

### Fluorescence microscopy of transfected cells and immunofluorescence

Cells were seeded onto 1 cm diameter glass coverslips in 24-well plates and transfected with expression vectors of interest, as described previously. Forty eight hours after transfection, cells were fixed in Histofix (Trend Scientific, Inc), and DNA was stained with Hoechst 33342 (Invitrogen). For immunofluorescence experiments, COS-7 cells were transfected as above, using the FOXL2-GFP and the SUMO1-His6 constructs. Forty eight hours after transfection, cells were washed with PBS, fixed for 15 min with Histofix (Trend Scientific, Inc), permeabilized for 15 min with PBS/1%Triton-X/10%FBS, and then blocked with 5% nonfat milk and 2% FBS in PBS/0.1%Tween 20. Cells were incubated overnight in the indicated antibody. Cells were then washed three times in PBS, and incubated for 1 h in an appropriate secondary antibody (diluted 1/500). In all cases, the coverslips were mounted on glass slides with DakoCytomaton (Dako). Cells were visualized by epifluorescence microscopy using a Leitz Aristoplan microscope, with a Leitz objective lens ×100/1.37 Oil with a numerical aperture of 0.17. Image acquisition was done with a JVC KX-F70B camera. We used the following antibodies with the indicated dilution: chicken anti-pan-PML ([51] a generous gift from Prof. H. de Thé) 1/500, mouse anti-SUMO1 (Zymed) 1/500, mouse anti-HSV tag (Novagen) 1/1000, mouse anti-SC35 (Sigma) 1/2000, rabbit anti-RNA Helicase A (Abcam) 1/1000, mouse anti-Pol2 (Covance) 1/1000, mouse anti-2,2,7-trimethylGuanosine cap (Calbiochem) 1/100.

### Fluorescence Recovery After Photobleaching (FRAP) experiments

FRAP experiments were conducted as previously [47]. Photobleaching experiments were performed 48 hours after transfection of COS-7 cells using a LEICA TCS SP2 AOBS confocal microscope. A small section of the nucleoplasm and of a nuclear body (when relevant) were bleached, and 50 images were collected every 1.635 s after bleaching. Data were collected from 10 to 15 different cells for each condition. Image analysis was performed with ImageJ software. Values for t1/2 are given ±SEM.

## Supporting Information

Figure S1 **FRAP experiments on COS-7 nuclei transfected with FOXL2-GFP alone and SUMO1-FOXL2-GFP.** For each condition: the leftmost panel shows GFP signal prior to bleaching. The second panel shows GFP signal immediately after bleaching (t = 1 s). The bleached portion(s) appears as dark regions. Black arrowheads indicate bleached nucleoplasmic portions and white arrowheads the bleached nuclear subcompartment. The two other panels show fluorescence recovery after 10 and 30 s. Complete recovery was achieved in all cases.
(TIF)

Figure S2 **SUMO-FOXL2 Bodies are not enriched in SC-35, Pol2, RNA Helicase A or TMG-capped RNA.** COS-7 cells were transfected with FOXL2-GFP and SUMO1. A representative cell with SUMO-FOXL2 Bodies is shown. SC-35 (i), RNA HA (ii), Pol2 (iii) and TMG-capped RNAs (iv) localization were detected by immunofluorescence, and DNA by Hoechst 33342 staining, pointing to an absence of colocalisation with SUMO-FOXL2 Bodies. This excludes the hypothesis that SUMO-FOXL2 Bodies are splicing factories, nucleoli, Cajal Bodies, gems or transcription factories.
(TIF)

Figure S3 **Spectrum attribution of the tryptic peptide EPEPPPSPGK in its unmodified (upper panel) and phosphorylated form (lower panel).** Proeminent neutral loss fragments corresponding to loss of H3P04, H3P04+H2O, H3P04+NH3 or H3P04+H2O+NH3 are present in the second spectrum (in green). Fragments attributed to the y-series of ions in the first spectrum (in blue) are conserved in the second spectrum (with the exception of y5) and bear the 80 Da modification, making it possible to localize it in the Pro-Pro-Ser sugfragment (y3 does not bear the modification as y6 does). Fragments attributed to the b-series of ions in the first spectrum (in red), most of them affected by an H2O neutral loss are also well conserved and confirm the localisation of the modification. Indeed b2 to b6 fragments are unaffected in size, whereas b8 is found with a −18 Da weight due to loss of an H2O group along with the phosphate (+80 Da–98 Da). This again localises the modification in the Pro-Ser subfragment and confirms the attribution.
(TIF)

Figure S4 **Spectrum attribution of the chymotryptic peptide** **AAAAAAAAAAAAAAGPGSPGAAAVVKGL** **in its unmodified (upper panel) and phosphorylated form (lower panel).** Neutral loss fragments characteristic of th phosphorylation are present but with pour abundance (in green). Spectra are extremely complex due to the size of the peptide but many peaks are attributed giving these peptides very high scores (XCorr = 6.97 for the upper spectrum, XCorr = 7.16 for the lower one) and little doubt about their attribution. Most peaks of the y-series (in blue) bearing the modification are present and well conserved between spectra, which allows to localize the modification in the Pro-Gly-Ser subfragment (y10 does not bear the modification, y13 does), further confirming the attribution.
(TIF)

Figure S5 **Spectrum attribution of the chymotryptic peptide** **LNNSWPLPQPPSPMPY** **in its unmodified (upper panel) and phosphorylated form (lower panel).** Neutral loss fragments are detected (in green) but are very minor. Peaks of the y-series (in blue) y7, y9, y11 and y12 are well conserved and bear the 80 Da modification in the second spectrum, allowing to localize it in the Pro-Pro-Ser-Pro-Met-Pro-Tyr subfragment. The b-series of ions are well conserved between spectra but give little informations about the modification position. Only small peaks attributed to y2 and b14 that are conserved between spectra suggest that the serine may more likely bear the modification (indeed y2 appears unshifted whereas b14 is shifted by 80 Da). However the intensity of this peaks does not allow to strictly conclude that serine 211 is the modified residue in this case.
(TIF)

Figure S6 **Spectrum attribution of the chymotryptic peptide** **SPASPATAAPPAPAPTSAPGLQF** **in its unmodified (upper panel) and phosphorylated form (lower panel).** Some neutral loss fragment (in green) characteristic of phosphorylation may be observed, but are once again of very low abundance. The fragmentation pattern is overall rather poor, but the main peaks are well conserved between spectra, notably y14 and y15 (in blue) and b9 and b14 (in red). This allows to localize the modification in the Ser-Pro-Ala-Ser-Pro-Ala-Thr-Ala-Ala subfragment, which still contains three possible sites for phosphorylation. Indeed both b9 and b14 are detected with both a +80 Da and a −18 Da shift in the second spectrum, whereas y14 and y15 are unshifted. A small peak attributed to y19 and unshifted between the spectra suggests that the modification is localised in the Ser-Pro-Ala-Ser subfragment, eliminating Thr329 as a phosphorylated residue, but this peak has a too low intensity to strictly conclude. According to the GPS 2.1 phospohrylation prediction tool, Ser326 is the residue most likely to be phosphorylated.
(TIF)

Figure S7 **Spectrum attribution of the chymotryptic peptide** **ASYPEPEDAAGAL** **in its unmodified (upper panel) and sulfated form (lower panel).** b-series of ions (in red) are well conserved between the two spectrum, with a 80 Da shift in the second spectrum. The doubly charged ions of the b-series are however much more intense in the second spectrum, compared to the monocharged ions. For example the intensity ratio of b122+ over b12+ is close to 3 in the first spectrum, and close to 30 in the second one. Other ions or other spectra are coherent with this observation. The y-series of ions is only represented by two proeminent peaks (in blue) in the first spectrum, corresponding to fragmentations before the two prolines of the peptide. This suggests that one of the peptide positive charges is solvated N-terminally of prolines, favoring fragmentation at these points, which is coherent with the higher pKa of proline amino function, compared to other amino acids. These two peaks completely disappear in the second spectrum, suggesting that the modification sequesters the positive charge, presumably through hydrogen bonding between the sulphate oxygens and carbonyl groups of the primary chain. This is also coherent with the prevalence of doubly-charged fragments in the second spectrum. One additional peak, attributed to y7+-NH3 by MASCOT appears in the second spectrum. Comparison with other spectra shows that this attribution is erroneous and that this fragment bears two positive charges. It may be due to an unusual c-ion, corresponding to the neutral loss of the last amino-acid.
(TIF)

Figure S8 **Spectrum attribution of the tryptic peptide** **KGWQNSIR** **in its unmodified (upper panel) and acetylated form (lower panel).** The monocharged y-series of ions (in blue) is well conserved between spectra and goes from y1 to y7, all peaks being unshifted, which localize the 42 Da mass shift to the first amino acid of the sequence, which indeed is a lysine. The b-series of ions (in red) are much less intense, but the most intense peaks of the first spectrum (b2, b6, b7) are found in the second spectrum shifted by 42 Da, confirming the presence of an acetyl group on the N-terminal lysine of this peptide.
(TIF)

Figure S9 **Spectrum attribution of the chymotryptic peptide** **TLDPACEDMFEKGNY** **in its unmodified (upper panel) and acetylated form (lower panel).** Most intense peaks of the y-series (in blue) and b-series (in red) are well conserved between the two spectra but do not allow for a very precise localisation of the modification. A more intense acetylated precursor could help obtaining more detailed fragmentation spectra and confirm the localisation of the modification.
(TIF)

Figure S10 **Spectrum attribution of the chymotryptic peptide** **DHDSKTGALHSRLDQGQF** **in its unmodified (upper panel) and acetylated form (lower panel).** The monocharged y-series of ions (in blue) allows to localize the 42 Da modification in the Asp-His-Asp-Ser-Lys subfragment as peaks corresponding to y9 to y13 fragment are well defined and unshifted between spectra. A more intense acetylated precursor could help obtaining more detailed fragmentation spectra and confirm the localisation of the modification.on lysine 366.
(TIF)

Table S1 **List of all peptides matches identified by Mascot and SEQUEST search engines.** Peptide matches attributed to FOXL2 and presenting a peptide score higher than 20 (for Mascot) or have a XCorr>2 when peptide charge was +2, >2.5 with peptide charge +3, >3 with peptide charge +4, and >4 with peptide charge >+5 (for SEQUEST) are listed in first (for Mascot) and second (for SEQUEST) worksheet. The predicted peptide modification, score, charge, precursor m/z, precursor MH+, predicted-to-measured mass difference (ΔM), column retention time and origin sample are indicated for each peptide match. Note that sulfation was not included as a possible modification, since search engines do not take into account the precursor mass and so can not distinguish between phosphorylation and sulfation. However, attribution of a phosphorylation on serine 4 or tyrosine 5 was systematically associated to a ΔM close to −9 mmu, corresponding to the difference between phosphate and sulfonate group.
(XLS)
