# Supplementary figures and images for: SUMOylation of the Forkhead Transcription Factor FOXL2 Promotes Its Stabilization/Activation through Transient Recruitment to PML Bodies
Source: PLoS One. 2011 Oct 12;6(10):e25463. doi: 10.1371/journal.pone.0025463 (PMC3192040; doi:10.1371/journal.pone.0025463)

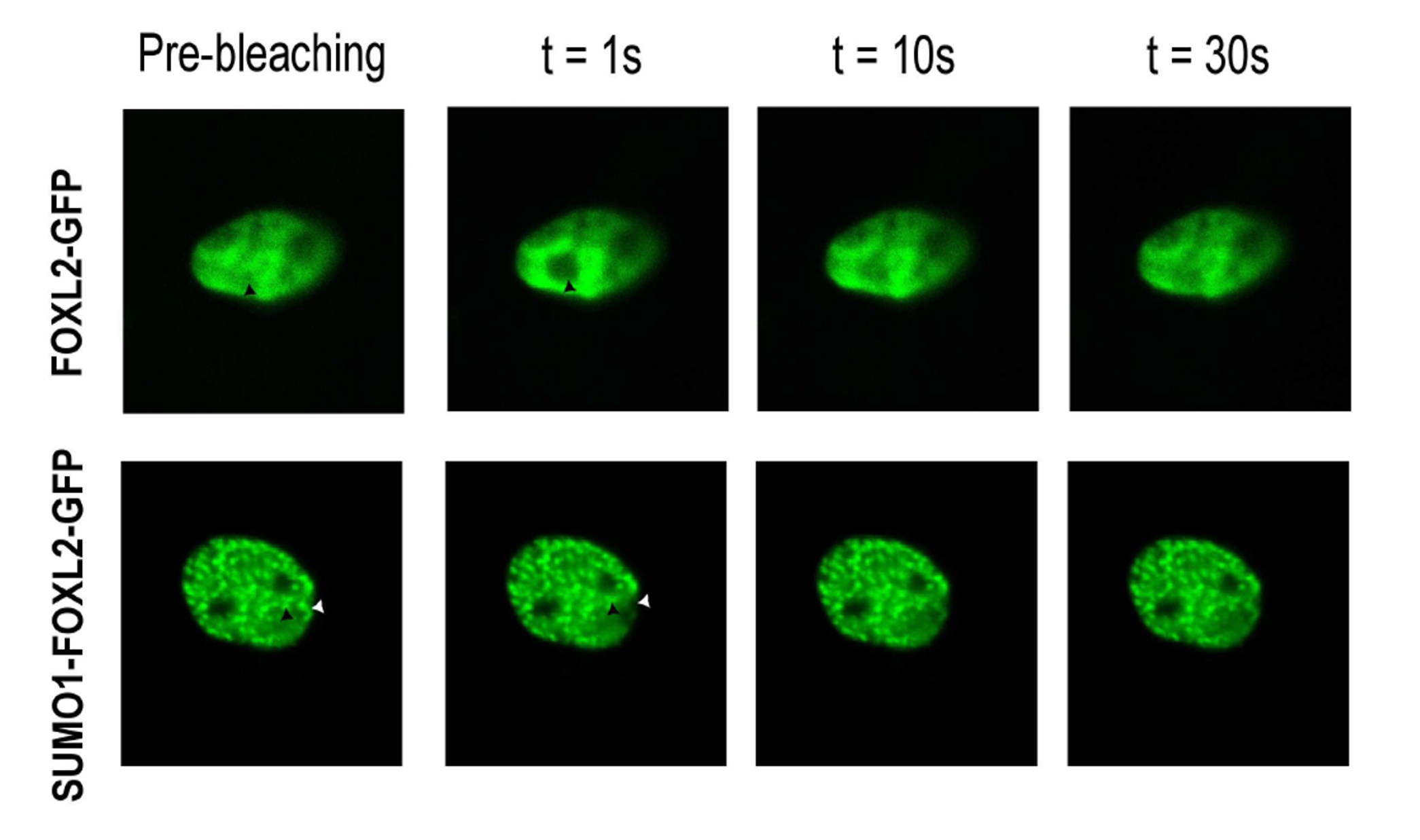

Supplement: Figure S1 — FRAP experiments on COS-7 nuclei transfected with FOXL2-GFP alone and SUMO1-FOXL2-GFP. For each condition: the leftmost panel shows GFP signal prior to bleaching. The second panel shows GFP signal immediately after bleaching (t = 1 s). The bleached portion(s) appears as dark regions. Black arrowheads indicate bleached nucleoplasmic portions and white arrowheads the bleached nuclear subcompartment. The two other panels show fluorescence recovery after 10 and 30 s. Complete recovery was achieved in all cases. (TIF) [file pone.0025463.s001.tif]

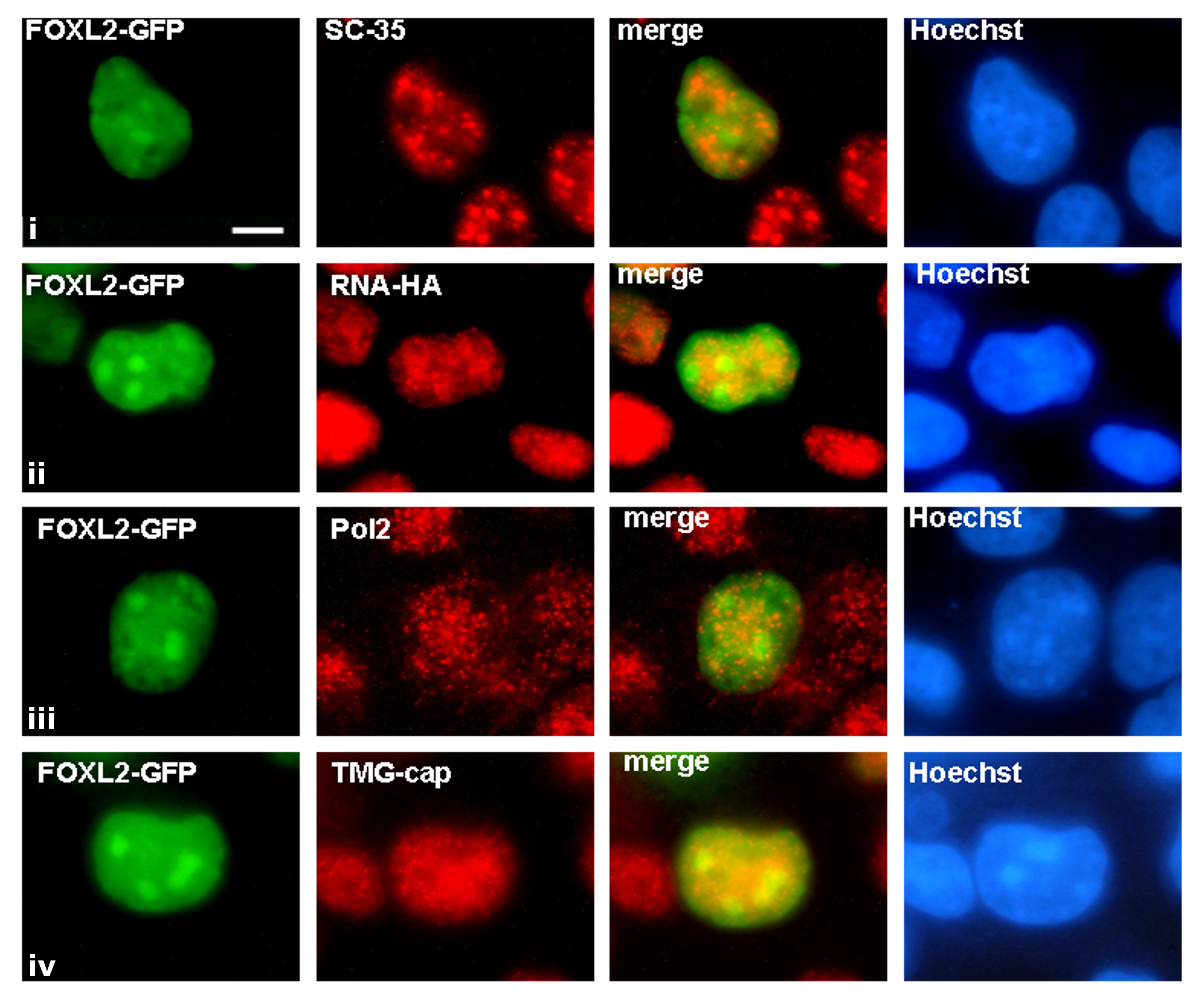

Supplement: Figure S2 — SUMO-FOXL2 Bodies are not enriched in SC-35, Pol2, RNA Helicase A or TMG-capped RNA. COS-7 cells were transfected with FOXL2-GFP and SUMO1. A representative cell with SUMO-FOXL2 Bodies is shown. SC-35 (i), RNA HA (ii), Pol2 (iii) and TMG-capped RNAs (iv) localization were detected by immunofluorescence, and DNA by Hoechst 33342 staining, pointing to an absence of colocalisation with SUMO-FOXL2 Bodies. This excludes the hypothesis that SUMO-FOXL2 Bodies are splicing factories, nucleoli, Cajal Bodies, gems or transcription factories. (TIF) [file pone.0025463.s002.tif]

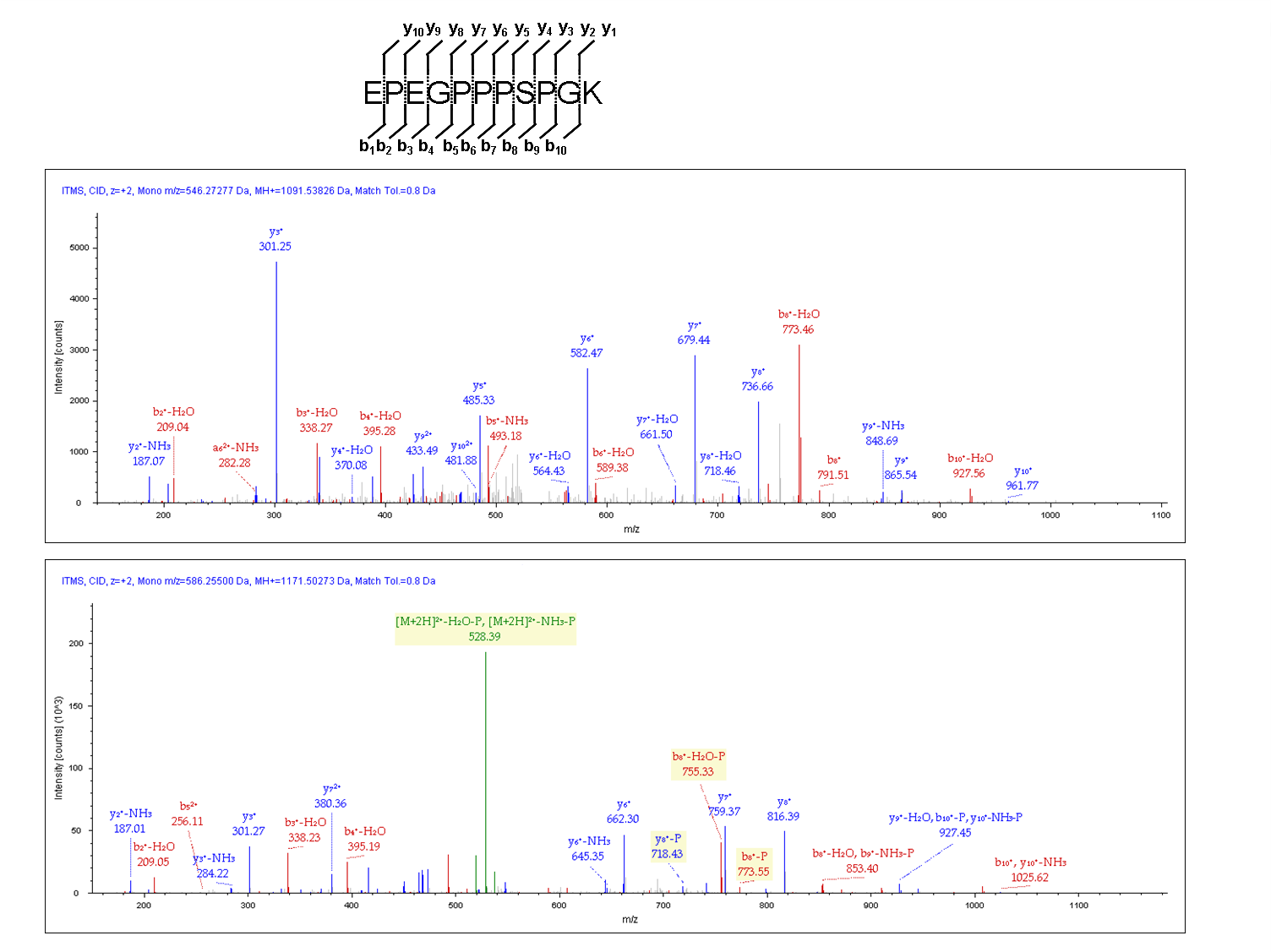

Supplement: Figure S3 — Spectrum attribution of the tryptic peptide EPEPPPSPGK in its unmodified (upper panel) and phosphorylated form (lower panel). Proeminent neutral loss fragments corresponding to loss of H3P04, H3P04+H2O, H3P04+NH3 or H3P04+H2O+NH3 are present in the second spectrum (in green). Fragments attributed to the y-series of ions in the first spectrum (in blue) are conserved in the second spectrum (with the exception of y5) and bear the 80 Da modification, making it possible to localize it in the Pro-Pro-Ser sugfragment (y3 does not bear the modification as y6 does). Fragments attributed to the b-series of ions in the first spectrum (in red), most of them affected by an H2O neutral loss are also well conserved and confirm the localisation of the modification. Indeed b2 to b6 fragments are unaffected in size, whereas b8 is found with a −18 Da weight due to loss of an H2O group along with the phosphate (+80 Da–98 Da). This again localises the modification in the Pro-Ser subfragment and confirms the attribution. (TIF) [file pone.0025463.s003.tif]

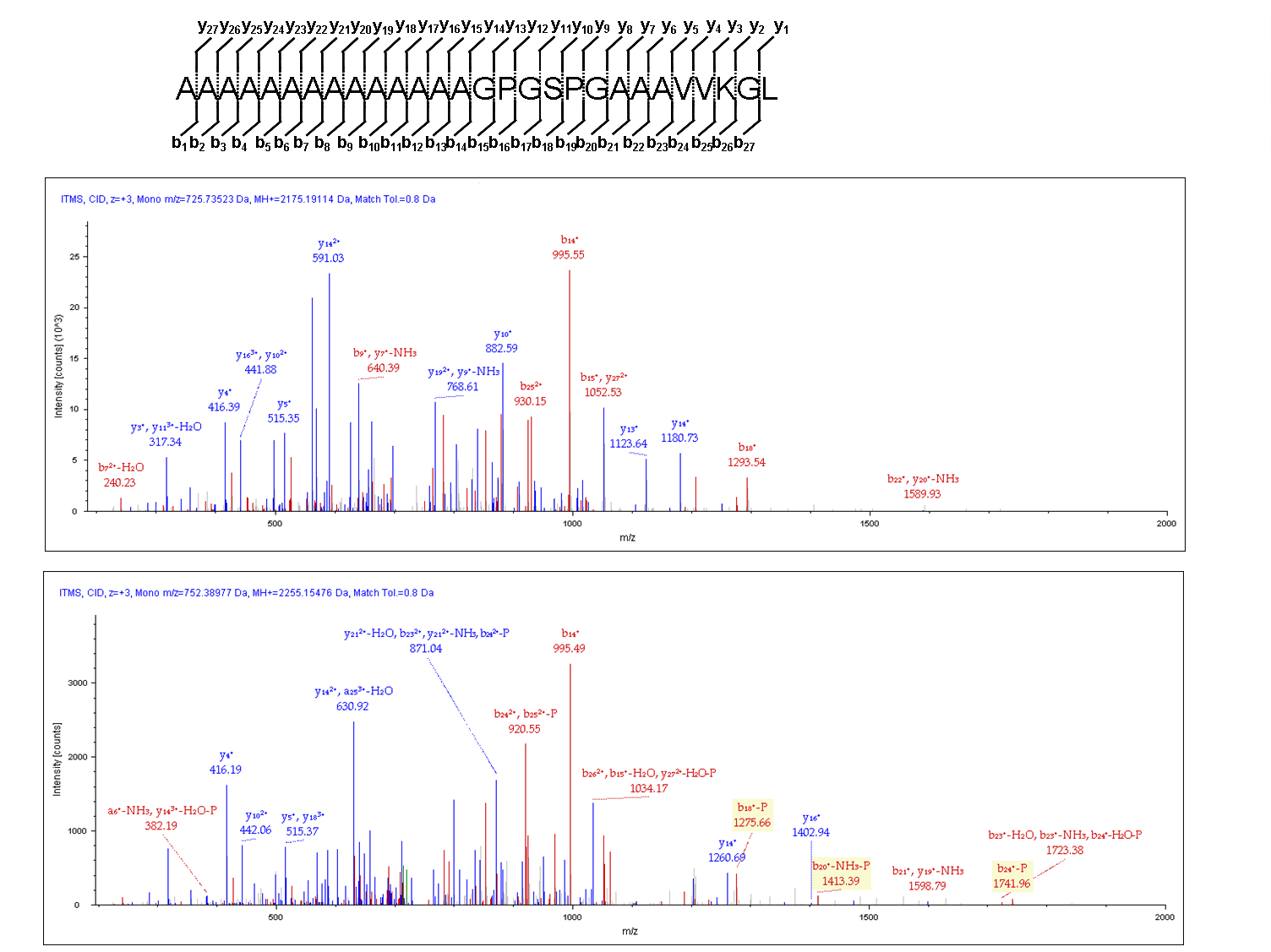

Supplement: Figure S4 — Spectrum attribution of the chymotryptic peptide AAAAAAAAAAAAAAGPGSPGAAAVVKGL in its unmodified (upper panel) and phosphorylated form (lower panel). Neutral loss fragments characteristic of th phosphorylation are present but with pour abundance (in green). Spectra are extremely complex due to the size of the peptide but many peaks are attributed giving these peptides very high scores (XCorr = 6.97 for the upper spectrum, XCorr = 7.16 for the lower one) and little doubt about their attribution. Most peaks of the y-series (in blue) bearing the modification are present and well conserved between spectra, which allows to localize the modification in the Pro-Gly-Ser subfragment (y10 does not bear the modification, y13 does), further confirming the attribution. (TIF) [file pone.0025463.s004.tif]

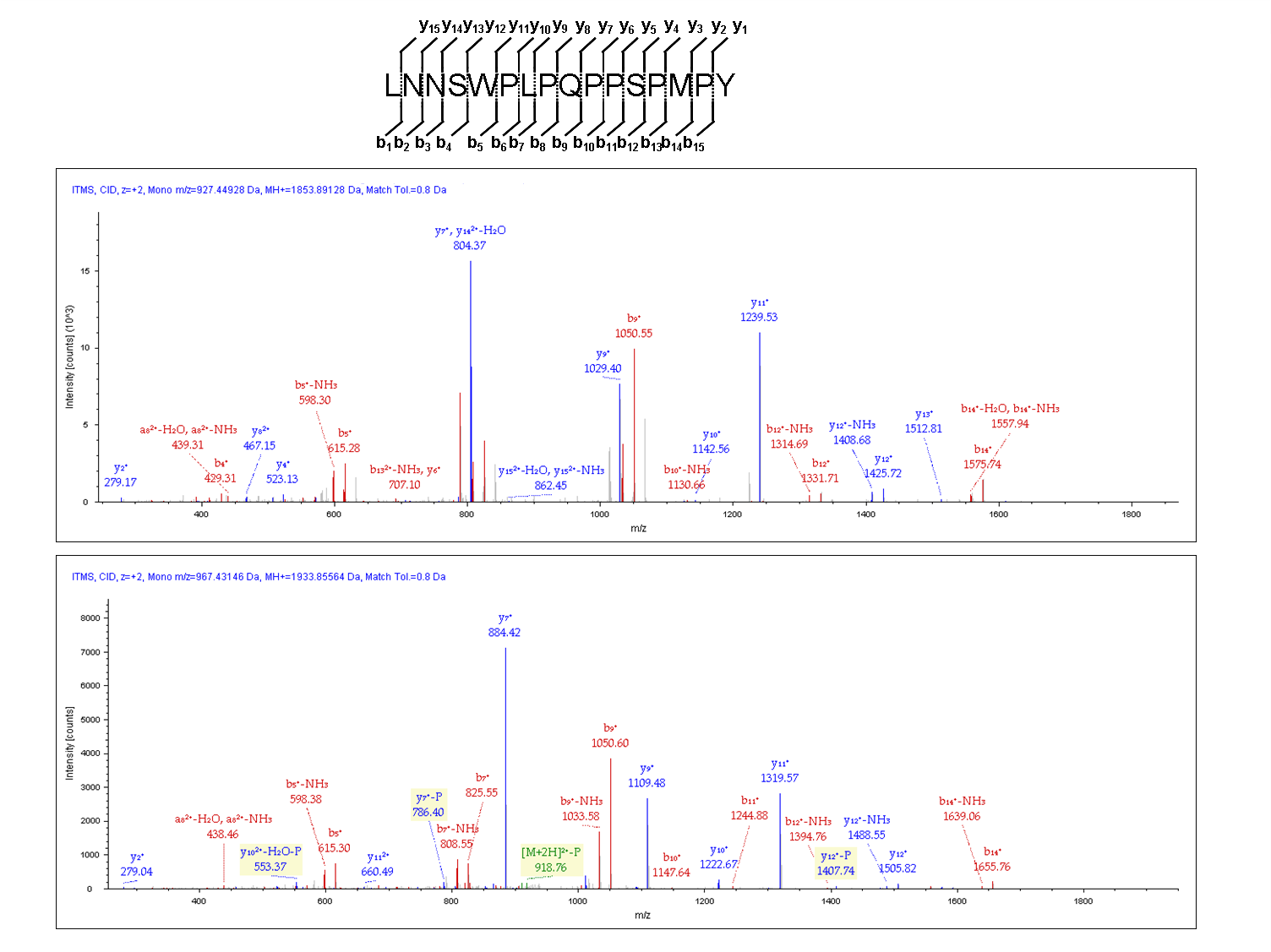

Supplement: Figure S5 — Spectrum attribution of the chymotryptic peptide LNNSWPLPQPPSPMPY in its unmodified (upper panel) and phosphorylated form (lower panel). Neutral loss fragments are detected (in green) but are very minor. Peaks of the y-series (in blue) y7, y9, y11 and y12 are well conserved and bear the 80 Da modification in the second spectrum, allowing to localize it in the Pro-Pro-Ser-Pro-Met-Pro-Tyr subfragment. The b-series of ions are well conserved between spectra but give little informations about the modification position. Only small peaks attributed to y2 and b14 that are conserved between spectra suggest that the serine may more likely bear the modification (indeed y2 appears unshifted whereas b14 is shifted by 80 Da). However the intensity of this peaks does not allow to strictly conclude that serine 211 is the modified residue in this case. (TIF) [file pone.0025463.s005.tif]

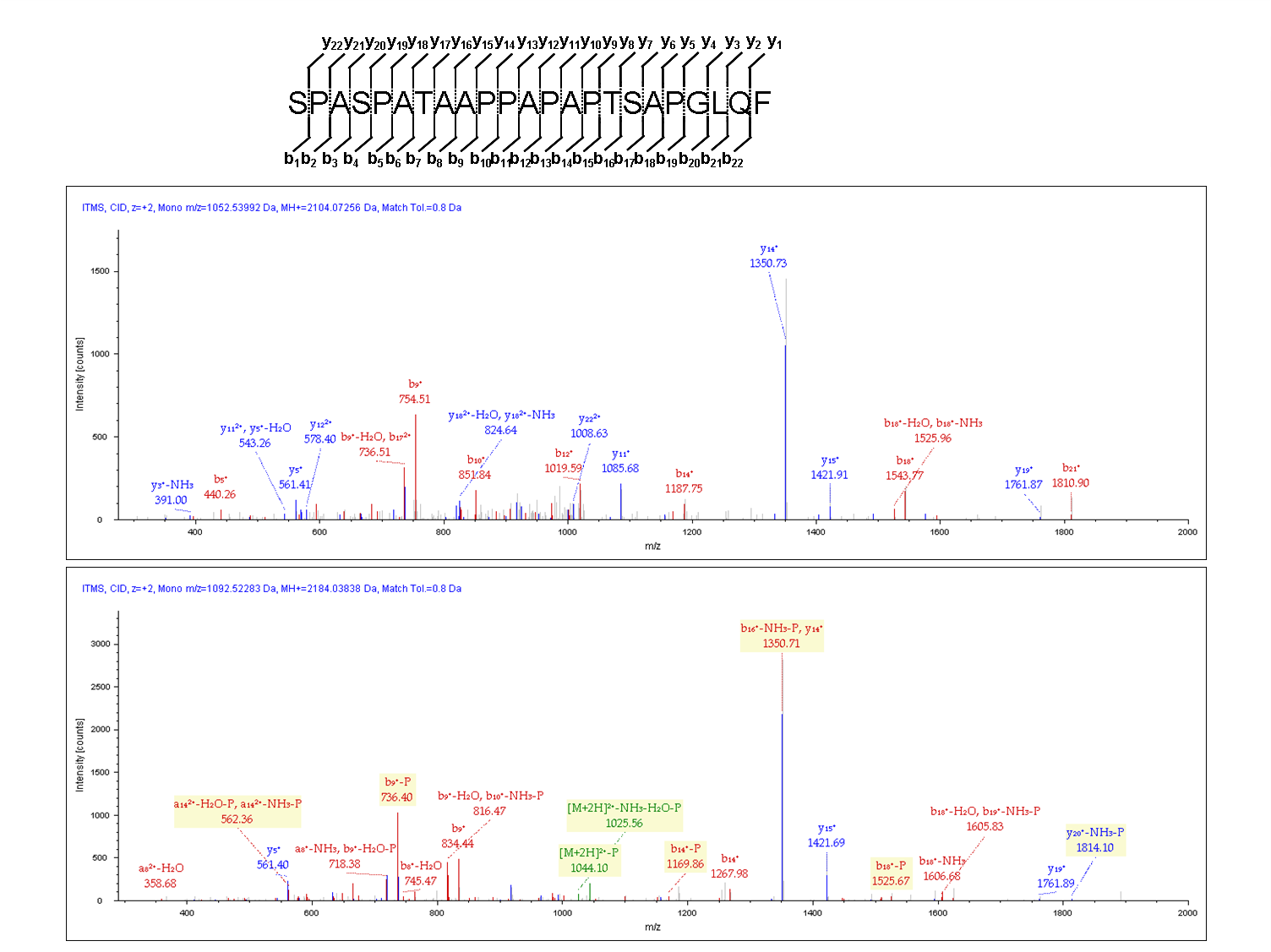

Supplement: Figure S6 — Spectrum attribution of the chymotryptic peptide SPASPATAAPPAPAPTSAPGLQF in its unmodified (upper panel) and phosphorylated form (lower panel). Some neutral loss fragment (in green) characteristic of phosphorylation may be observed, but are once again of very low abundance. The fragmentation pattern is overall rather poor, but the main peaks are well conserved between spectra, notably y14 and y15 (in blue) and b9 and b14 (in red). This allows to localize the modification in the Ser-Pro-Ala-Ser-Pro-Ala-Thr-Ala-Ala subfragment, which still contains three possible sites for phosphorylation. Indeed both b9 and b14 are detected with both a +80 Da and a −18 Da shift in the second spectrum, whereas y14 and y15 are unshifted. A small peak attributed to y19 and unshifted between the spectra suggests that the modification is localised in the Ser-Pro-Ala-Ser subfragment, eliminating Thr329 as a phosphorylated residue, but this peak has a too low intensity to strictly conclude. According to the GPS 2.1 phospohrylation prediction tool, Ser326 is the residue most likely to be phosphorylated. (TIF) [file pone.0025463.s006.tif]

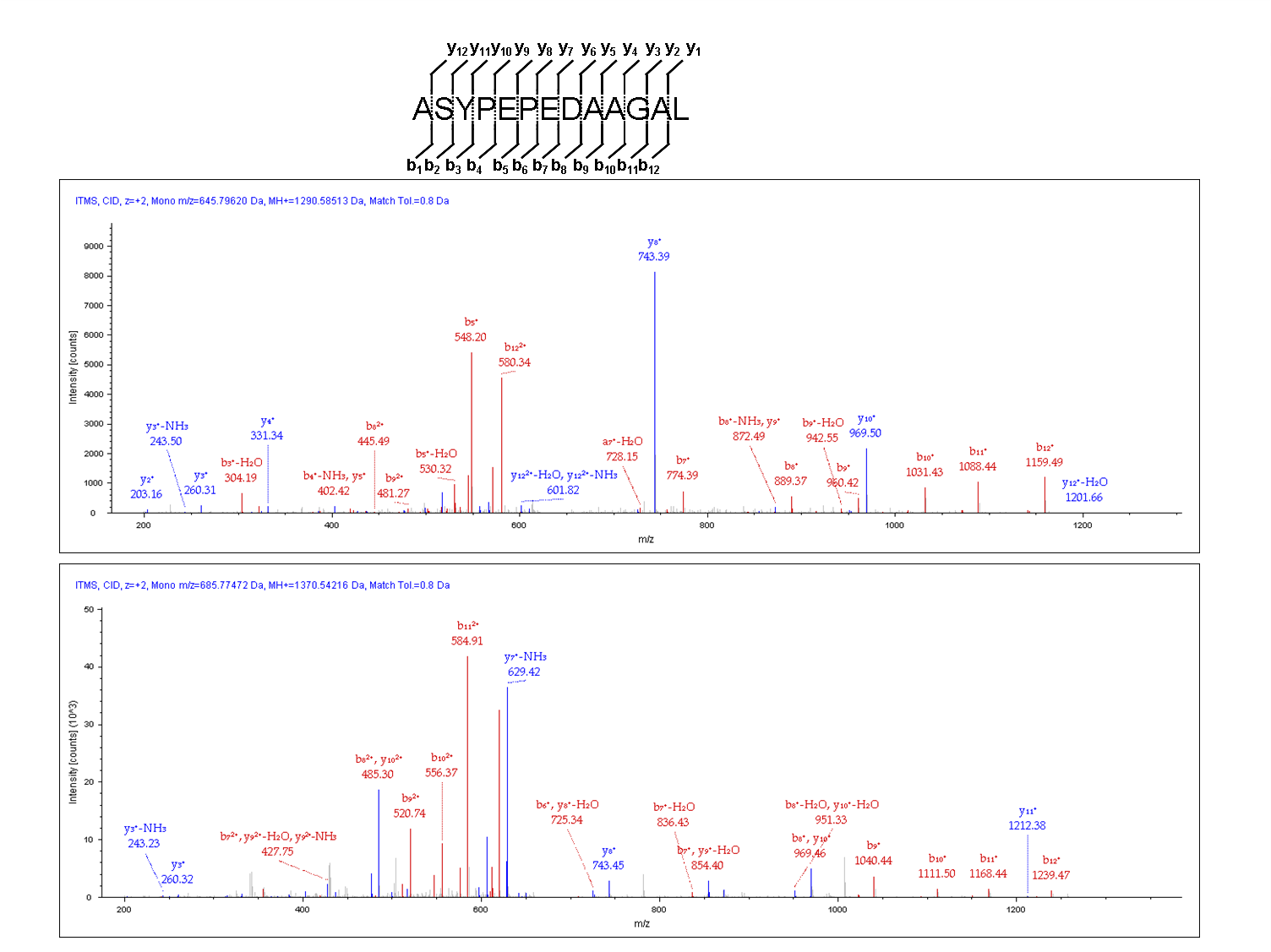

Supplement: Figure S7 — Spectrum attribution of the chymotryptic peptide ASYPEPEDAAGAL in its unmodified (upper panel) and sulfated form (lower panel). b-series of ions (in red) are well conserved between the two spectrum, with a 80 Da shift in the second spectrum. The doubly charged ions of the b-series are however much more intense in the second spectrum, compared to the monocharged ions. For example the intensity ratio of b122+ over b12+ is close to 3 in the first spectrum, and close to 30 in the second one. Other ions or other spectra are coherent with this observation. The y-series of ions is only represented by two proeminent peaks (in blue) in the first spectrum, corresponding to fragmentations before the two prolines of the peptide. This suggests that one of the peptide positive charges is solvated N-terminally of prolines, favoring fragmentation at these points, which is coherent with the higher pKa of proline amino function, compared to other amino acids. These two peaks completely disappear in the second spectrum, suggesting that the modification sequesters the positive charge, presumably through hydrogen bonding between the sulphate oxygens and carbonyl groups of the primary chain. This is also coherent with the prevalence of doubly-charged fragments in the second spectrum. One additional peak, attributed to y7+-NH3 by MASCOT appears in the second spectrum. Comparison with other spectra shows that this attribution is erroneous and that this fragment bears two positive charges. It may be due to an unusual c-ion, corresponding to the neutral loss of the last amino-acid. (TIF) [file pone.0025463.s007.tif]

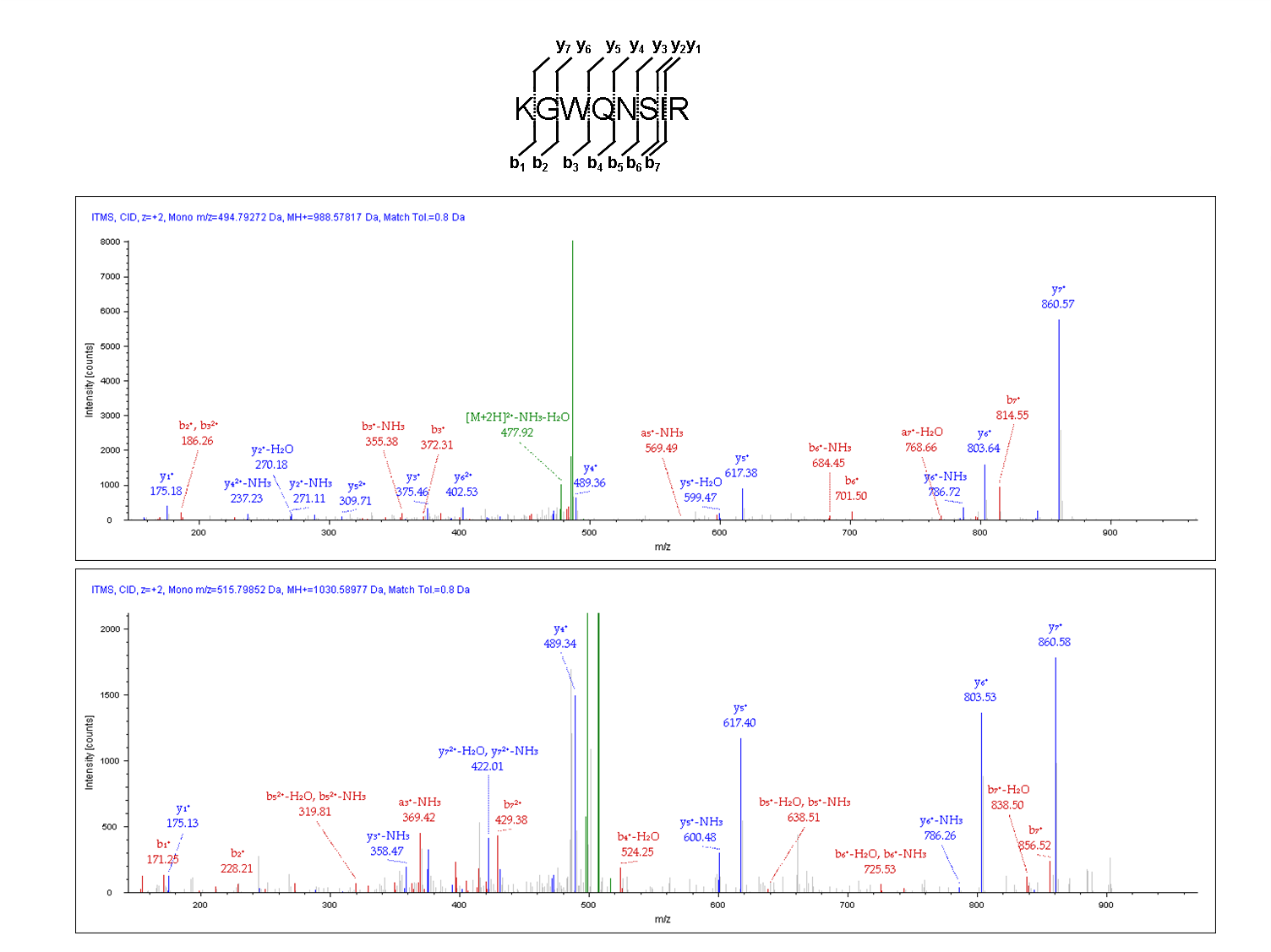

Supplement: Figure S8 — Spectrum attribution of the tryptic peptide KGWQNSIR in its unmodified (upper panel) and acetylated form (lower panel). The monocharged y-series of ions (in blue) is well conserved between spectra and goes from y1 to y7, all peaks being unshifted, which localize the 42 Da mass shift to the first amino acid of the sequence, which indeed is a lysine. The b-series of ions (in red) are much less intense, but the most intense peaks of the first spectrum (b2, b6, b7) are found in the second spectrum shifted by 42 Da, confirming the presence of an acetyl group on the N-terminal lysine of this peptide. (TIF) [file pone.0025463.s008.tif]

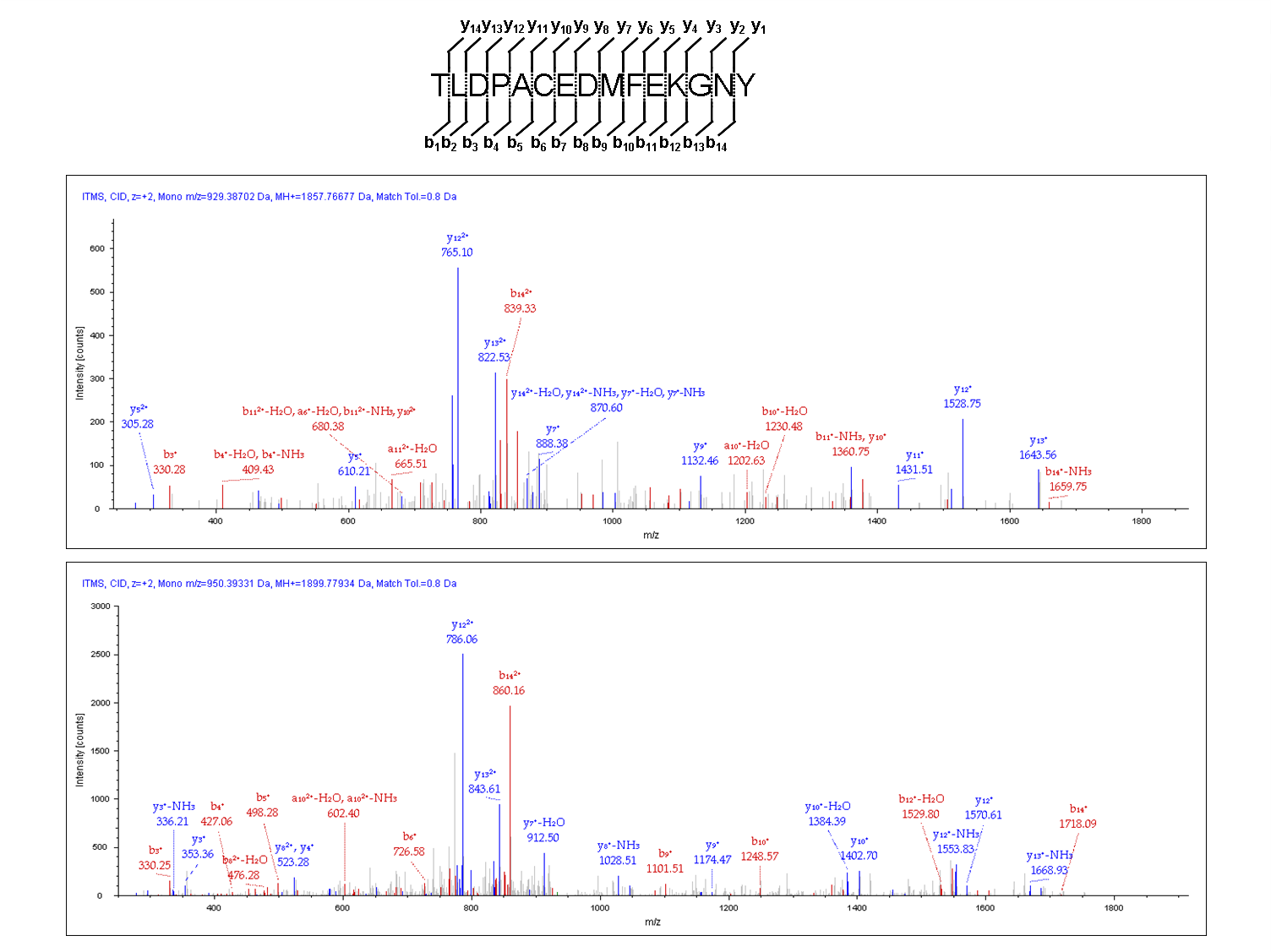

Supplement: Figure S9 — Spectrum attribution of the chymotryptic peptide TLDPACEDMFEKGNY in its unmodified (upper panel) and acetylated form (lower panel). Most intense peaks of the y-series (in blue) and b-series (in red) are well conserved between the two spectra but do not allow for a very precise localisation of the modification. A more intense acetylated precursor could help obtaining more detailed fragmentation spectra and confirm the localisation of the modification. (TIF) [file pone.0025463.s009.tif]

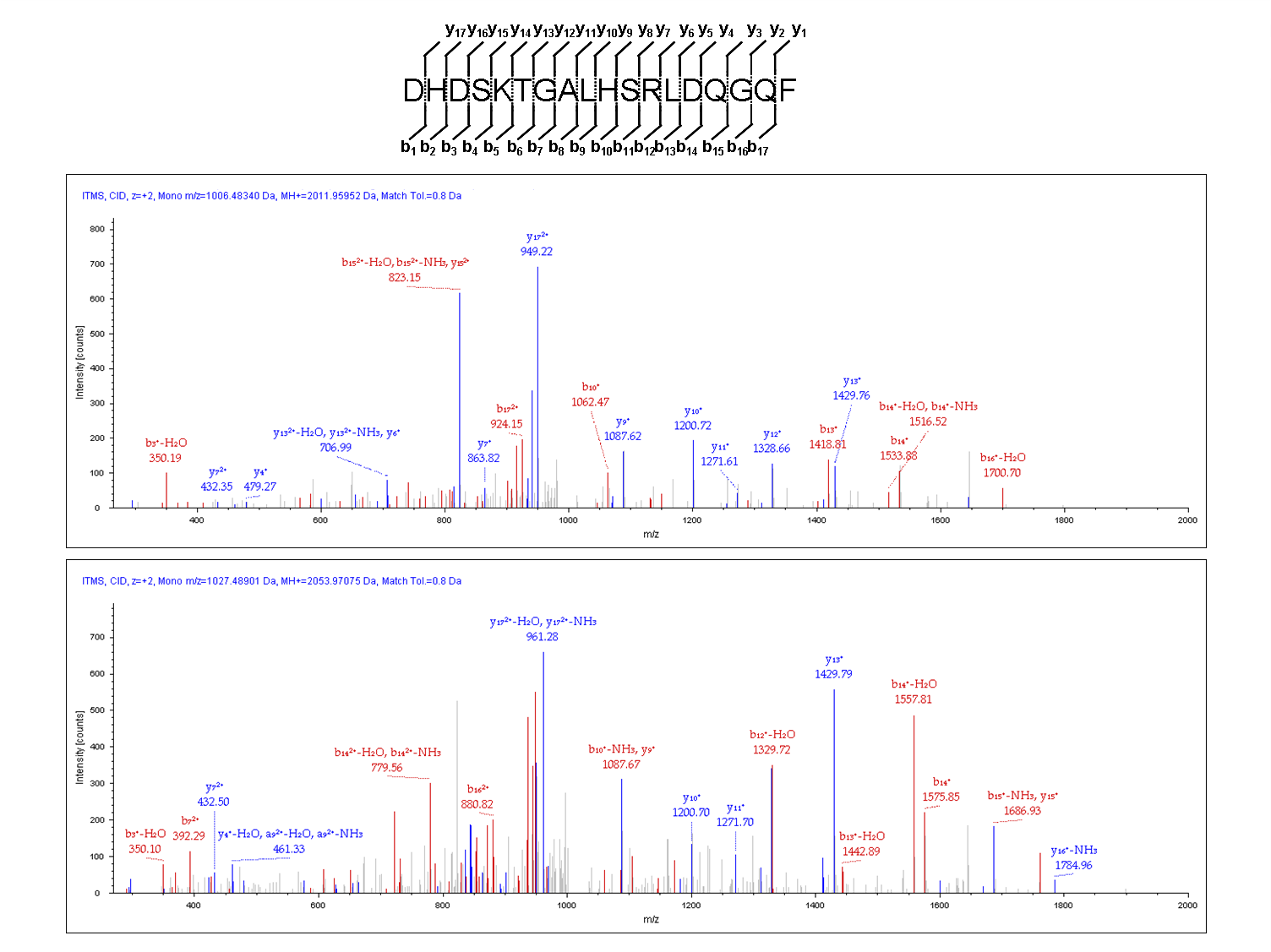

Supplement: Figure S10 — Spectrum attribution of the chymotryptic peptide DHDSKTGALHSRLDQGQF in its unmodified (upper panel) and acetylated form (lower panel). The monocharged y-series of ions (in blue) allows to localize the 42 Da modification in the Asp-His-Asp-Ser-Lys subfragment as peaks corresponding to y9 to y13 fragment are well defined and unshifted between spectra. A more intense acetylated precursor could help obtaining more detailed fragmentation spectra and confirm the localisation of the modification.on lysine 366. (TIF) [file pone.0025463.s010.tif]
